# Systems analysis identifies miR-29b regulation of invasiveness in melanoma

**DOI:** 10.1186/s12943-016-0554-y

**Published:** 2016-11-16

**Authors:** Miles C. Andrews, Joseph Cursons, Daniel G. Hurley, Matthew Anaka, Jonathan S. Cebon, Andreas Behren, Edmund J. Crampin

**Affiliations:** 1 Olivia Newton-John Cancer Research Institute, Heidelberg, VIC 3084 Australia; 2 Ludwig Institute for Cancer Research, Melbourne-Austin Branch, Cancer Immunobiology Laboratory, Heidelberg, VIC 3084 Australia; 3 School of Cancer Medicine, La Trobe University, Heidelberg, VIC 3084 Australia; 4 Department of Medicine, University of Melbourne, Parkville, VIC 3010 Australia; 5 Systems Biology Laboratory, University of Melbourne, Parkville, VIC 3010 Australia; 6 ARC Centre of Excellence in Convergent Bio-Nano Science, University of Melbourne, Parkville, VIC 3010 Australia; 7 School of Mathematics and Statistics, University of Melbourne, Parkville, VIC 3010 Australia; 8 Centre for Systems Genomics, University of Melbourne, Parkville, VIC 3010 Australia; 9 Department of Medicine, University of Toronto, Toronto, ON Canada

**Keywords:** Systems biology, Cancer, micro-RNA, Melanoma, Phenotypic switching, Statistical association, TargetScan, DIANA-microT CDS

## Abstract

**Background:**

In many cancers, microRNAs (miRs) contribute to metastatic progression by modulating phenotypic reprogramming processes such as epithelial-mesenchymal plasticity. This can be driven by miRs targeting multiple mRNA transcripts, inducing regulated changes across large sets of genes. The miR-target databases TargetScan and DIANA-microT predict putative relationships by examining sequence complementarity between miRs and mRNAs. However, it remains a challenge to identify which miR-mRNA interactions are active at endogenous expression levels, and of biological consequence.

**Methods:**

We developed a workflow to integrate TargetScan and DIANA-microT predictions into the analysis of data-driven associations calculated from transcript abundance (RNASeq) data, specifically the mutual information and Pearson’s correlation metrics. We use this workflow to identify putative relationships of miR-mediated mRNA repression with strong support from both lines of evidence. Applying this approach systematically to a large, published collection of unique melanoma cell lines – the Ludwig Melbourne melanoma (LM-MEL) cell line panel – we identified putative miR-mRNA interactions that may contribute to invasiveness. This guided the selection of interactions of interest for further in vitro validation studies.

**Results:**

Several miR-mRNA regulatory relationships supported by TargetScan and DIANA-microT demonstrated differential activity across cell lines of varying matrigel invasiveness. Strong negative statistical associations for these putative regulatory relationships were consistent with target mRNA inhibition by the miR, and suggest that differential activity of such miR-mRNA relationships contribute to differences in melanoma invasiveness. Many of these relationships were reflected across the skin cutaneous melanoma TCGA dataset, indicating that these observations also show graded activity across clinical samples. Several of these miRs are implicated in cancer progression (miR-211, -340, -125b, −221, and -29b). The specific role for miR-29b-3p in melanoma has not been well studied. We experimentally validated the predicted miR-29b-3p regulation of LAMC1 and PPIC and LASP1, and show that dysregulation of miR-29b-3p or these mRNA targets can influence cellular invasiveness in vitro.

**Conclusions:**

This analytic strategy provides a comprehensive, systems-level approach to identify miR-mRNA regulation in high-throughput cancer data, identifies novel putative interactions with functional phenotypic relevance, and can be used to direct experimental resources for subsequent experimental validation.

Computational scripts are available: http://github.com/uomsystemsbiology/LMMEL-miR-miner

**Electronic supplementary material:**

The online version of this article (doi:10.1186/s12943-016-0554-y) contains supplementary material, which is available to authorized users.

## Background

Phenotypic switching is an important process that facilitates melanoma progression, metastasis, and resistance to therapy [1–6]. Despite the neural crest (i.e. non-epithelial) origin of melanocytes, melanomas display cadherin-switching and functional behaviours that resemble epithelial-to-mesenchymal plasticity (EMP). Melanomas often harness lineage-specific molecular pathways from more primitive (less-differentiated) states in order to switch between proliferative, minimally-invasive (i.e. ‘epithelial-like’, or ‘E-like’) and invasive, minimally-proliferative (i.e. ‘mesenchymal-like’, or ‘M-like’) phenotypes [7–9]. Several transcription factors, including ZEB1/2, SNAIL1/2, TWIST1/2, MITF and JUN, have been shown to play key roles in melanoma EMP-like processes [4, 7, 9, 10]; however, the mechanisms by which internal and micro-environmental signals are integrated to modulate transcriptional activity are not fully understood.

Micro-RNAs (miRs) are short (18–24 nucleotide) non-coding RNAs which play an important role regulating the activity of other RNA transcripts. They have been implicated in the oncogenesis and progression of several cancers, as reviewed thoroughly elsewhere [11, 12]. Some miRs directly target important transcription factors, such as miR-200 regulation of ZEB in epithelial carcinomas [13, 14], while in melanoma, miR-148 mediated dysregulation of MITF [15] and miR-125b control of JUN [16] have been noted. In melanoma, a network of miRs, including miR-211 and miR-222 has been shown to mediate some effects of oncogenic BRAF signalling [17]. ArrayCGH studies suggest miR-29b copy number increases in melanoma and a number of miRs have been implicated in melanocyte transformation [18, 19], melanoma progression [20–23], modulating the extent and mode of melanoma cell invasiveness [17, 24, 25], and switching of cellular phenotype (i.e. epithelial-mesenchymal plasticity) in a number of epithelial cancers [14, 26, 27].

Identifying endogenous miRs that play a key role in oncogenesis remains challenging for a number of reasons. Firstly, within mammalian cells, miRs exert their effects through multiple mechanisms that are difficult to observe experimentally utilising any single current method. Multiple lines of evidence have shown that miRs regulate protein abundance by modulating protein translation [28], influencing mRNA transcript stability [29, 30] or through both effects [31]. These effects may not be entirely independent [32, 33], and it has been argued that changes in mRNA stability can be attributed to repression of translation [33]. A recent review has detailed the molecular mechanisms mediating target transcript sequestration or degradation, and translational inhibition [32].

Secondly, there is extensive ‘crosstalk’ and broader, coordinated targeting by miRs [27, 34], by virtue of the mapping of miRs to multiple potential transcript targets with potentially different binding affinities. One common effect which influences miR activity is variation in the abundance of alternative RNA targets within the intracellular milieu – although the effect of protein-coding competitive endogenous RNAs (ceRNAs) is thought to be minimal [35], non-coding ceRNAs [36] and circular RNAs [37, 38] have been shown to act as miRNA “sinks” which exert biological effects in a combinatorial fashion. Together, these effects make it difficult to identify individual miR-mRNA interactions which are consistent across many experimental systems and conditions.

Finally, well-known differences between cellular processes active in transformed cell lines and in primary tissues taken from patients lead to a differing repertoire of miRs, transcripts, and metabolic intermediates. As a direct consequence, experimental results observed in one model system, particularly in cell lines, may not be observable in or relevant to patient tumours in vivo [39]. Overcoming these challenges requires an integrative systems analysis relating miR abundance to target mRNA abundance, as well as to a relevant phenotype. Here we report such an analysis using the Ludwig Melbourne melanoma (LM-MEL) cell line panel [40], an experimental resource designed for the identification and verification of molecular mechanisms contributing to the heterogeneity observed across melanoma tumours.

The LM-MEL panel data were collected from 57 established melanoma cell lines [40] which display a range of phenotypes across the epithelial-like to mesenchymal-like spectrum [41]. The original LM-MEL molecular data were supplemented with miR transcript abundance data (Additional file 1), and information on invasiveness through matrigel (Additional file 2), which allowed us to identify putative interactions between miRs and mRNAs associated with differences in the functional phenotypes of these melanoma cell lines.

We applied statistical measures of association (Pearson’s correlation [r_P_] and mutual information [MI]) together [42] to identify miRs and mRNAs which showed a strong inverse association across the LM-MEL dataset. Mutual information is an information-theoretic metric that measures divergence from statistical independence [43]. It contains a $\log \left(\frac{P\left(A,B\right)}{P(A)P(B)}\right)$ term that tends to zero with statistical independence, where *P*(*A*, *B*) = *P*(*A*)*P*(*B*). Mutual information and MI-based metrics have been successfully applied for the analysis of large biological data sets [44–46] (*further details within* Methods/Statistical associations). Conversely, Pearson’s correlation purely measures the tendency towards a linear association, although it does provide directionality through positive/negative values.

This report describes a systems approach to identify putative regulatory miR-mRNA relationships across related cell lines and clinical samples, applying measures of statistical association and refining the results through the principled inclusion of knowledge from specific relevant databases. Our computational workflow identifies a novel role for miR-29b-3p in regulating melanoma invasiveness, and the results improve our understanding of how miR activity can influence melanoma phenotype switching and EMP.

## Results

### Changes in miR abundance were predicted to regulate mRNA transcript stability across a panel of melanoma cell lines

**Fig. 1:**
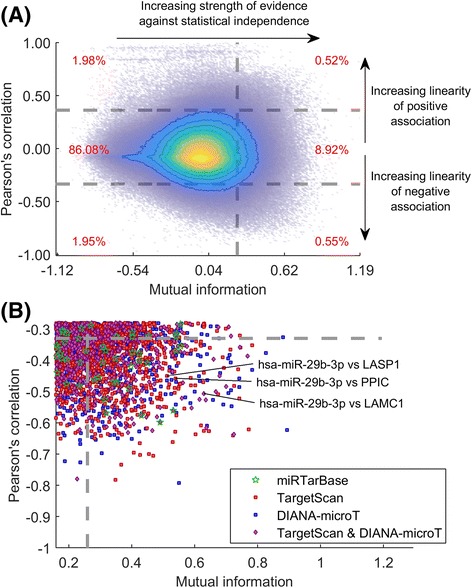
Systems analysis of database-enriched statistical associations between miR and mRNA transcripts. **a** Density and contour plot for mutual information and Pearson’s correlation between transcript abundance of all miR and mRNA pairs across the LM-MEL cell line panel (*n* = 57). Associations within the top 10% of mutual information values (*vertical dashed line*) and bottom (most negative) 2.5% of Pearson’s correlation values (*horizontal dashed lines*) were selected as those with strong negative associations (*relative fraction of associations in red*). **b** These associations (**a**, *bottom right*) were matched against high-confidence, predicted relationships from TargetScan and DIANA-microT, and previously validated relationships from miRTarBase. Relationships between hsa-miR-29b-3p and LAMC1, LASP1, and PPIC are examined below, Additional file 3 lists all putative relationships

Given the evidence implicating miRs in cancer progression and invasiveness, we hypothesised that miRs with differential activity across the LM-MEL cell line panel may drive post-transcriptional regulatory changes that influence cell phenotype. To identify relationships where miRs may regulate mRNA transcripts through reduced stability, we examined miR and mRNA transcript abundance across the LM-MEL cell line panel and selected those with a strong, negative (or inverse) statistical association between miR and mRNA abundance (Fig. 1a).

### Numerous known and predicted miR-mRNA interactions show evidence of post-transcriptional regulation across the LM-MEL cell line panel

We integrated TargetScan [47–49] and DIANA-microT [50, 51] (Additional file 1) to limit the search space for putative miR-mRNA interactions. Numerous strong, negative associations across the LM-MEL data were supported by sequence-based predictions with a relatively high confidence (TargetScan top 15^th^ percentile; and/or DIANA-microT top 30^th^ percentile; details in Methods/Databases). Matching putative relationships to miRTarBase (using ‘strong evidence’ experimental methods), only a small sub-set of interactions appear to have been previously validated (Fig. 1b). A full list of predicted relationships is given in Additional file 3 with further details.

### Putative regulatory associations contain a number of previously validated associations

**Fig. 2:**
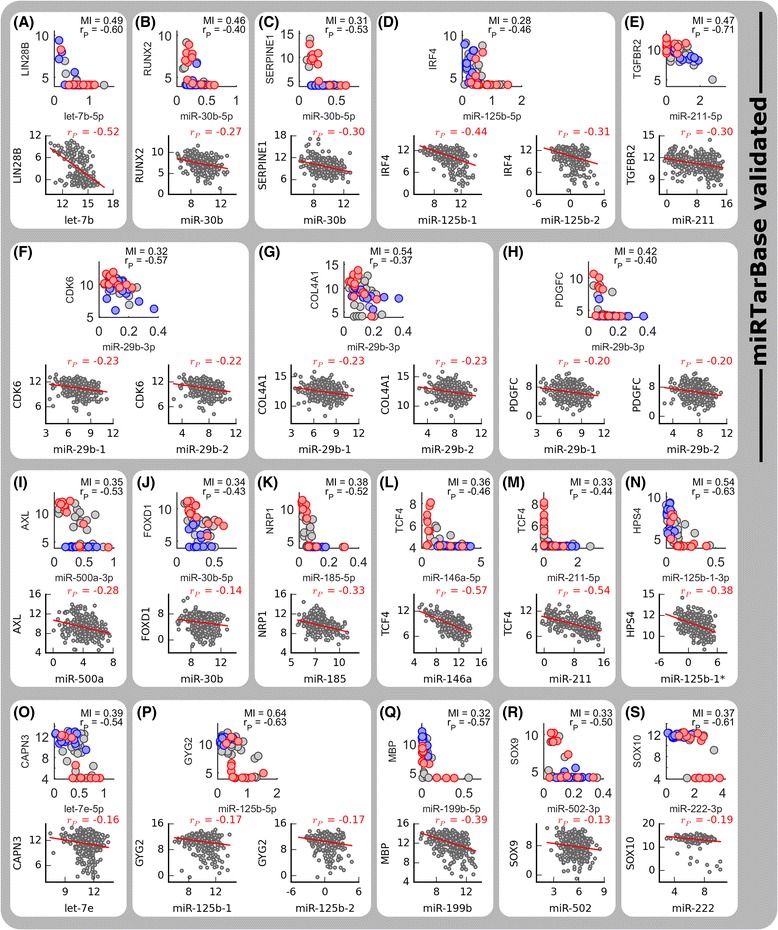
Many putative miR-mRNA relationships involve mRNAs previously implicated in EMP and melanoma phenotype switching. LM-MEL and TCGA (*top and bottom subplots, respectively*) transcript abundance data for (**a**-**h**) selected, previously validated relationships (miRTarBase, ‘strong experimental evidence’; *see* Methods/Databases), and (**i**-**q**) putative relationships regulating mRNAs implicated in melanoma phenotype switching by Widmer et al. [3]. Further details are given in Additional file 4. Predicted regulatory interactions involving SOX transcription factors integral to melanoma phenotype switching are shown (**r-s**).

Selected relationships are shown in Fig. 2 using in vitro LM-MEL data with phenotypic annotations (top of panel; red, high matrigel-invasiveness cell lines; blue, low invasiveness), and in vivo TCGA [52] Skin Cutaneous Melanoma (SKCM) data (bottom of panel).

Several putative relationships have been validated across different human cell systems (Fig. 2a-h; ‘miRTarBase validated’), and were supported by TCGA data (*further details within* Additional file 4). In parallel, a number of putative relationships emerged which have not been previously observed within human cell lines, and many of these potentially novel relationships involved mRNA transcripts implicated in melanoma phenotype switching [3] and invasive behaviours (Fig. 2i-q; Additional file 4).

Within the unvalidated interactions, the predicted regulatory interactions between the transcription factors SOX9 and miR-502-3p (Fig. 2r; LM-MEL r_P_ = −0.50, MI = 0.33; TCGA r_P_ = −0.13), and SOX10 and miR-222-3p (Fig. 2s; LM-MEL r_P_ = −0.61, MI = 0.37; TCGA r_P_ = −0.19), is particularly interesting. In melanoma, SOX10 functions both independently and in cooperation with MITF to promote more differentiated and/or proliferative cellular states [53, 54]. A SOX10-low state is associated with reduced cell proliferation and engagement of EMT-like processes in melanoma to promote more invasive phenotypes [55] - a state maintained, in part, through mutual-antagonism with the closely related transcription factor SOX9 [56]. SOX10 suppression contributes to BRAF- and/or MEK-inhibitor resistance in BRAF mutated melanoma, by activating TGFβ signalling to upregulate EGFR and PDGFRB [57], whilst increasing SOX9 transcript abundance has been observed in breast cancer EMT [58]. SOX9-high LM-MEL cell lines are also enriched for an invasive phenotype (Fig. 2r) and there is a distinct subset of SOX10-low, high-invasive LM-MEL cell lines (Fig. 2s) which appears to be recapitulated within the TCGA data.

### A number of miRs implicated in the progression of melanoma and other cancers were enriched for relationships with differential regulatory activity

**Fig. 3:**
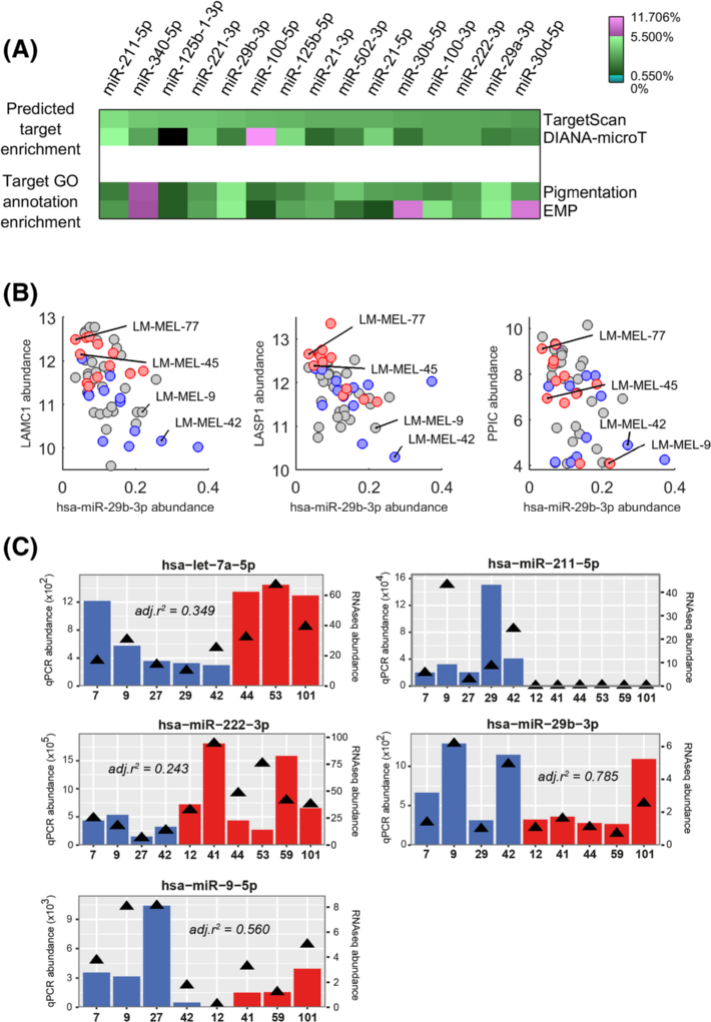
**a** Putative target list enrichment for individual miRs using TargetScan and DIANA-microT target lists (*first and second row, respectively*). Relative enrichment of combined miR target lists are also shown for GO terms associated with pigmentation or EMP. **b** Relationships between miR-29b-3p and LAMC1 (*left*), LASP1 (*centre*) and PPIC (*right*) within the LM-MEL data; for cell lines with matrigel invasiveness measurements, these data are overlaid (*red, high invasiveness; blue, low invasiveness*). **c** miR transcript abundances measured by quantitative PCR (qPCR; *bar graph*) for selected miRs and LM-MEL cell lines (indicated by number along the x-axis), plotted against high-throughput LM-MEL panel data (*black triangles*); adjusted regression coefficients between these datasets are indicated (negative for miR-211-5p due to multiple non-expressing cell lines)

As detailed earlier, miRs can drive phenotypic change through the coordinated regulation of several mRNA targets. To examine this we calculated the relative enrichment of ‘active associations’ (Fig. 1b) for each miR across the LM-MEL data. The top five miRs when using high confidence TargetScan lists were miR-211-5p, miR-340-5p, miR-125b-1-3p, miR-221-3p and miR-29b-3p (Fig. 3a, *top row*). High confidence DIANA-microT target lists also suggested differential activity for miR-100-5p across the LM-MEL cell line panel (Fig. 3a, *second row*).

The top four micro-RNAs identified have been shown previously to regulate melanoma cell proliferation, migration and/or invasiveness. As mentioned above, expression levels of miR-211 are inversely related to melanoma cell migration and invasion, and it has been shown to function as a tumour-suppressor through target genes including IGF2R, TGFBR2 (Fig. 2e) and NFAT5 [59, 60]. Similarly, miR-340 downregulation promotes melanoma progression [61] through de-repression of drug transporters [62], RAS-RAF-MAPK signalling components [63] and the key melanocytic transcription factor MITF [64]. The role of miR-125b in cancer progression has also been studied extensively – in melanoma miR-125b over-expression reduces cell proliferation and migration, mediated partly by the direct suppression of c-JUN mRNA translation [16]; however, much of the literature has examined miR-125b-5p (the dominant mature form; Fig. 3a, *seventh column*), rather than the -3p forms. Finally, miR-221 has a well-studied role in melanoma progression [20, 65] and circulating miR-221 has been proposed as a melanoma biomarker [66].

### Enrichment analyses implicate miR-29b-3p in melanoma phenotype switching

The role of the fifth ranked micro-RNA, miR-29b-3p, in melanoma has not yet been explored. In other cancer types, members of the miR-29a/b/c family influence EMP [67–69], and act as tumour suppressors [70–72]. In cutaneous melanoma, down-regulation of miR-29c has been associated with an adverse prognosis, attributed in part to its regulation of transcripts for the DNA methyltransferases DNMT3A/DNMT3B [73].

Given roles for phenotype switching [4, 74, 75] and pigmentation changes [76] in melanoma progression, we extracted Gene Ontology database terms associated with melanogenesis or pigmentation (‘*Pigmentation*’), or epithelial-mesenchymal plasticity (‘*EMP*’). Next, we examined the relative enrichment of these ontological classifications within high-confidence miR target lists from TargetScan and/or DIANA-microT (Fig. 3a, *at right*). Consistent with its role in melanoma progression [61–63], miR-340-5p had a particularly strong loading for both “pigmentation” and “EMP” categories, whilst miR-29b-3p was also enriched around 10-fold (~5.5%), providing further support that miR-29b-3p may be regulating melanoma cell invasiveness through a diverse repertoire of regulatory interactions.

TargetScan predicts hsa-miR-29-3p family-mediated regulation of Peptidylprolyl Isomerase C (Cyclophilin C; PPIC), Laminin, γ1 (LAMC1) and LIM and SH3 Protein 1 (LASP1), and DIANA-microT also predicts miR-29b-3p regulation of LAMC1 (Fig. 1b). All three predicted targets show a strong negative association across the LM-MEL cell line panel (Fig. 3b) in a manner suggesting that reduced expression of miR-29b-3p and increased expression of these targets is associated with more invasive cell lines (Fig. 3b).

### Validation of miR and mRNA transcript abundances across a subset of LM-MEL cell lines

We performed quantitative PCR (qPCR) on selected LM-MEL cell lines to validate the high-throughput panel expression data for interactions of interest. Using miRNA-specific qPCR, transcript abundance of hsa-let-7a-5p, hsa-miR-211-5p, hsa-miR-222-3p, hsa-miR-29b-3p and hsa-miR-9-5p within total RNA showed reasonable concordance with sequencing-derived abundance data from the LM-MEL panel, and expected trends were apparent for divergent miR abundances between cell lines classified as E-like or M-like (Fig. 3c). Expression levels of target gene transcripts assessed using standard qPCR confirmed measurable levels of ATP2A2, CPEB1, TCF4, NRP1, ADAM19, CAV2, LAMC1, LASP1 and PPIC in the cell lines studied, again with trends for divergent levels between E- and M-like cell lines consistent with microarray-derived data (Figure AF5.1 within Additional file 5).

### hsa-miR-29b-3p regulates LAMC1, LASP1 and PPIC within several LM-MEL cell lines

As shown in Fig. 2 (panels F-H), targets of hsa-miR-29b-3p (miR-29b) include several molecules with documented roles in extracellular matrix formation, sensing, signalling or modulation, consistent with a role in invasiveness or EMT-like processes. To elucidate the role of miR-29b in melanoma invasiveness, we examined the effect of miR-29b perturbation on the transcript abundance of several putative targets in a subset of LM-MEL cell lines. Cells were transiently transfected at high efficiency with either miR-29b inhibitor or mimic (Figure AF5.2A & B within Additional file 5). MiR-specific qPCR demonstrated a 2-3-log increase in detectable miR-29b levels following transfection with miR-29b mimic at 1nM (Figure AF5.2C within Additional file 5), whilst transfection of miR-29b inhibitor did not appreciably alter qPCR-detectable mature miR-29b abundance.

**Fig. 4:**
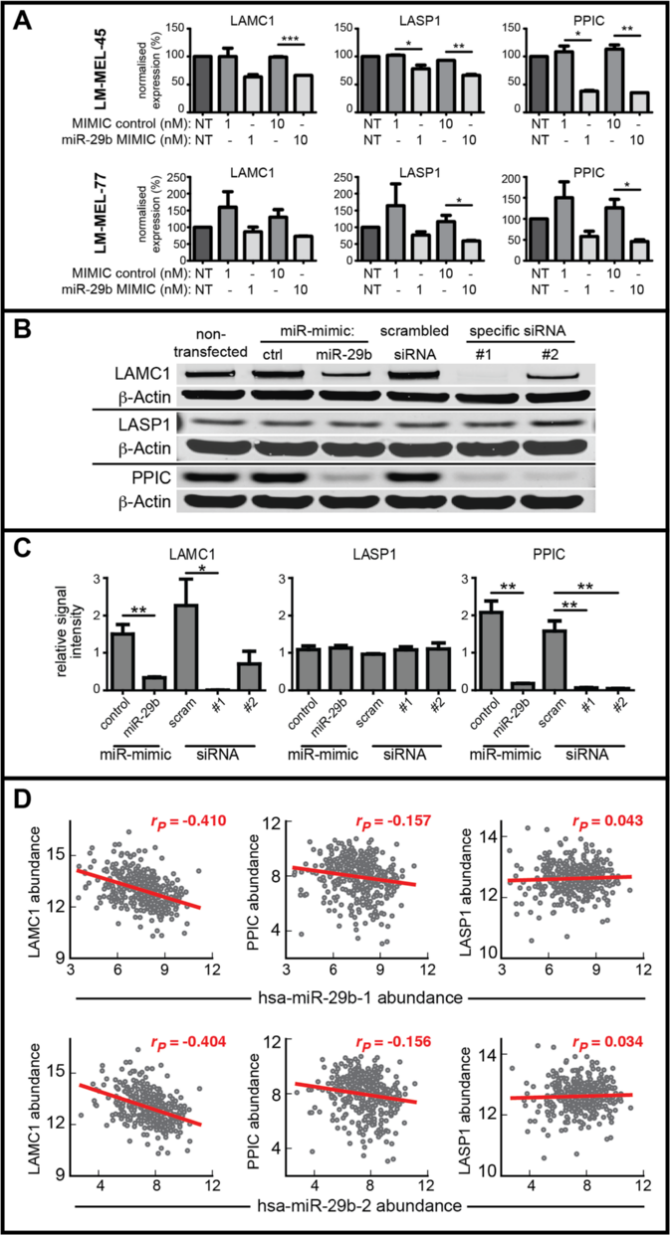
The effects of hsa-miR-29b transfection on mRNA transcript and protein levels for putative targets: LAMC1, LASP1 and PPIC. **a** For LM-MEL-45 and −77 (*upper and lower row, respectively*), two mesenchymal-like cell lines, mRNA transcript levels were measured following transfection with hsa-miR-29b mimic. Expression has been normalised to non-transfected (NT) controls. Data represent mean + SD from independent biological duplicates performed in technical triplicates. **b** Representative Western blots and (**c**) quantified densitometry for LM-MEL-45 cell lysate harvested 96 h post-transfection with 10nM of miR-29b mimic, miR mimic negative control (ctrl), siRNA scrambled (scram) control, or specific siRNA to the indicated mRNA target. Densitometry data represent mean + SEM signal intensities for independent triplicates normalised against a β-actin loading control, and shown relative to non-treated cells. Pairwise one-tailed *t*-test statistics are indicated by * *p* < 0.05, ** *p* < 0.01, *** *p* < 0.001. **d** Relationships between miR-29b-1 and miR-29b-2 (immature -3p precursors), and LAMC1 (*at left*) LASP1 (*at centre*) and PPIC (*at right*) within TCGA SKCM data. Pearson’s correlations and the line of best fit are shown in *red*

MiR-29b inhibitor treatment of the E-like cell lines LM-MEL-9 and LM-MEL-42 (high baseline levels of miR-29b) induced dose-dependent increases in transcript abundance of ADAM19, CAV2, LAMC1, PPIC, and to a lesser degree, LASP1 (Figure AF5.3 within Additional file 5). Treatment of the M-like cell lines LM-MEL-45, −57 and −77 with a miR-29b mimic markedly reduced transcript abundance of ADAM19, LAMC1, and PPIC, with more modest effects on CAV2 and LASP1 (Fig. 4a and Figure AF5.3 within Additional file 5). All three genes selected for further investigation (LAMC1, LASP1 and PPIC) exhibited a dose-dependent reduction in transcript level following miR-29b mimic transfection, although this effect was near-maximal at 1nM.

We assessed the effect of miR-29b overexpression on protein levels for putative targets using Western blotting for LAMC1, LASP1 and PPIC in the prototypical M-like cell line LM-MEL-45. Marked reductions in LAMC1 and PPIC protein levels were seen at 96 h following transient transfection with a miR-29b mimic, consistent with the effects of specific siRNAs (Fig. 4b & c). Contrasting transcript abundance changes, LASP1 protein levels were unaffected by either the miR-29b mimic or specific siRNA treatment, suggesting high protein stability and minimal LASP1 protein turnover within the duration of the assay (Fig. 4b & c).

To examine whether these putative relationships are present in vivo, we examined matched miR and mRNA transcript abundances within TCGA skin and cutaneous melanoma (SKCM) data. The abundances of mature hsa-miR-29b-1 and hsa-miR-29b-2 transcripts were examined against mRNA abundances for LAMC1, PPIC, and LASP1 (Fig. 4d). Within the TCGA data there was a relatively strong negative association between LAMC1 and miR-29b, no association between miR-29b and LASP1, and a weak negative association between miR-29b and PPIC.

### hsa-miR-29b-3p reduces melanoma cell motility and invasiveness through mechanisms beyond single-gene targets

Mild effects were observed for miR-29b-3p mimic in reducing LM-MEL-45 outgrowth survival at 21 days, with little change in cellular proliferation observed at 72 h (Figure AF5.4 & AF5.5 within Additional file 5).

**Fig. 5:**
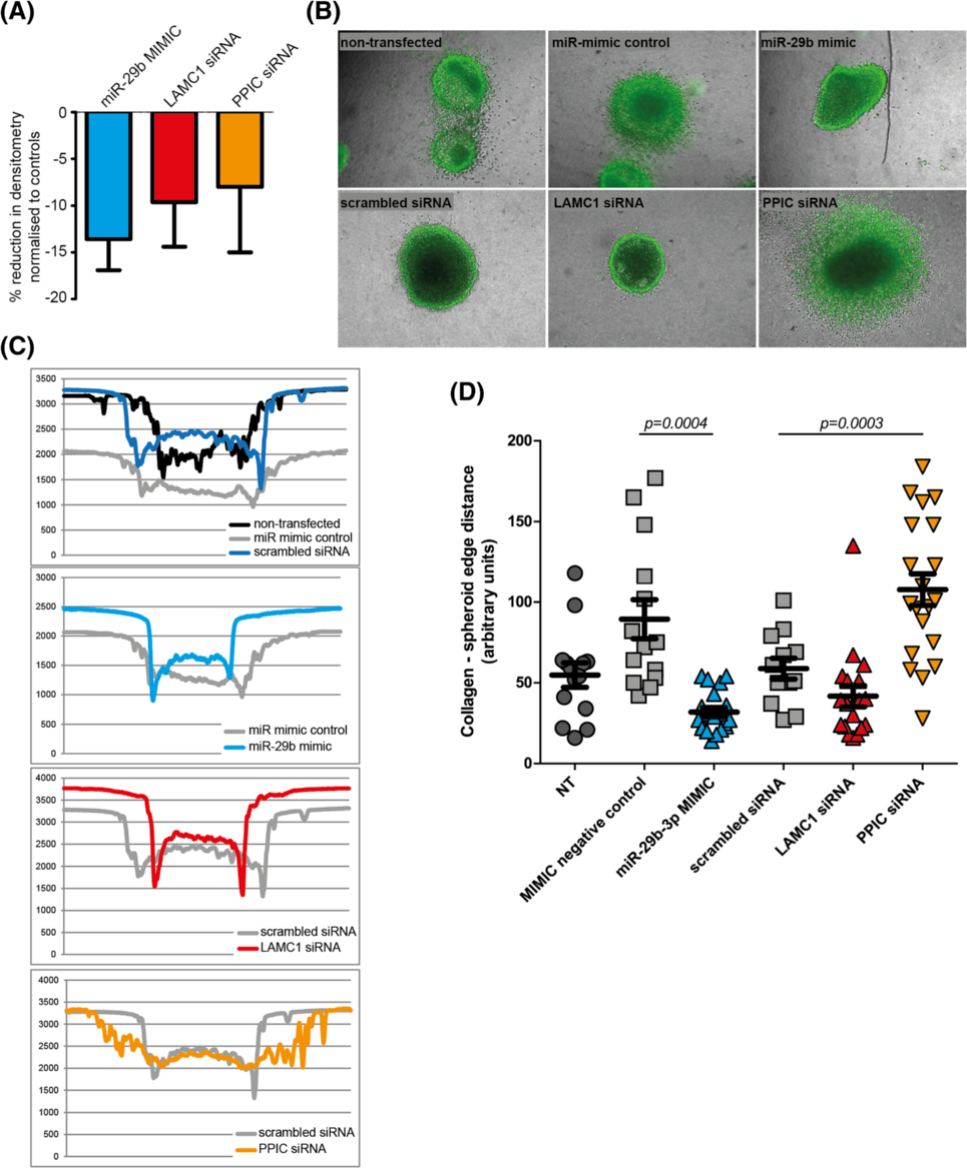
Effects of miR-29b, LAMC1 and PPIC perturbation on motility and invasion of LM-MEL-45. **a** In the absence of a chemotactic gradient, migration of LM-MEL-45 cells into a central detection zone was decreased by treatment with a miR-29b-3p mimic, and partially phenocopied by siRNA-mediated knock-down of either LAMC1 or PPIC (data represent mean + SEM relative to controls of independent triplicates, all treatments at 10nM). **b** Following reverse transfection with the indicated agents (final concentrations all 10nM), and overnight culture on agarose, LM-MEL-45 spheroids were embedded into a bovine collagen type I matrix and allowed to invade over 24 h prior to staining with a *green*-fluorescent viable cell dye and imaging (representative spheroids shown). Treatment with a miR-29b-3p mimic virtually ablated any invasion of cells into surrounding collagen, whilst siRNA-mediated knockdown of PPIC led to marked invasion and/or lack of cohesion, seen as a pronounced spheroid halo. **c** Cross-sectional cellular density measurements of representative spheroids (see also Figure AF5.6 within Additional file 5) confirm relative lack of invasive cells at the spheroid surface in miR-29b-3p mimic treated cells (*second panel*) and, to a lesser extent, in LAMC1 knockdown cells (*third panel*). PPIC knockdown cells displayed a broad spheroid cell density profile consistent with extensive cellular egress into surrounding collagen (*bottom panel*). **d** Quantitation of the invasion distance from spheroid edge confirmed significant decrease in collagen invasion following miR-29b-3p overexpression, and increase following PPIC knockdown. *Error bars* show mean ± SEM of at least seven spheroids per treatment

To better assess the effect of miR-29b on melanoma cell motility, cultured LM-MEL-45 cells were allowed to migrate into a central detection zone of the Oris plate assay system (without a chemotactic gradient) following treatment with miR-29b mimic or specific siRNA for either LAMC1 or PPIC. LASP1 was not examined on the basis that we did not demonstrate reduced levels of protein within the duration of this assay. The density of cells migrating into the central detection zone was measured after 48 h. Following all three treatments, LM-MEL-45 cells demonstrated a comparable reduction in migration, compared with control-treated cells (Fig. 5a).

Next, spheroid collagen invasion assays were performed to compare the same treatments in a three-dimensional matrix-embedded setting. Spheroids were imaged following staining for viable cells. As predicted, miR-29b treatment reduced LM-MEL-45 cellular invasion into surrounding collagen almost entirely (representative spheroids, Fig. 5b). Invasiveness was generally less in siRNA-treated cells, with minimal difference seen for LAMC1 knock-down. Cross-sectional cellular density profiles (Fig. 5c; *method illustrated by* Figure AF5.6 within Additional file 5) and quantitation of the collagen invasion distance (Fig. 5d) confirmed sharp transitions between relatively acellular surrounding collagen matrix and cell spheroid following miR-29b mimic and LAMC1 transfection (Fig. 5c), consistent with reduced invasion of cells into surrounding collagen.

Unexpectedly, siRNA-mediated knockdown of PPIC dramatically increased LM-MEL-45 cell invasiveness, and on cross-sectional spheroid cell density analysis, no clear transition point was observed in spheroids treated with PPIC siRNA (Fig. 5b & c), indicating diffuse cellular invasion into surrounding collagen. Within the limitations of the assay system used, it could not be determined whether this diffuse invasion of PPIC-knocked-down cells represented enhanced cellular invasiveness, decreased cell-cell cohesion, or a mixture of both processes. The effect of PPIC knockdown was opposite to that predicted following the observation that miR-29b-3p overexpression, which reduces PPIC expression, led to a decrease in spheroid collagen invasion. This contradictory effect is entirely consistent with the notion that microRNAs exert their overall observable effects through the summation of effects on multiple individual gene targets. Such individual effects may be concordant, or discordant, with the overall effect. In this case, specific PPIC knockdown appears to be pro-invasive, but when combined with the totality of perturbations induced by miR-29b-3p overexpression, the pro-invasive effect of PPIC knockdown is more than negated, leading to a net reduction in invasion. This finding demonstrates that the effects of miR-29b-3p on melanoma cell migration are not accurately replicated by perturbation of any single mRNA target in isolation.

## Discussion

Integrated analysis of matched microRNA and mRNA abundance data across a large panel of melanoma cell lines identified several putative regulatory relationships influencing melanoma phenotypic plasticity. We demonstrate a novel modulatory effect for miR-29b-3p activity on melanoma cell invasiveness, most likely mediated by the combined effects on multiple gene targets. Dysregulation of miR-29b has been observed in a range of carcinomas of gastrointestinal [69, 71], breast [68], gynaecologic [77] and prostatic origin [78], with a more specific role in suppression of tumour growth and metastasis in colorectal cancer cells shown to involve blockade of epithelial-to-mesenchymal transition [69]. In breast cancer models, it has been shown that miR-29b is induced by, and mediates the EMT-inhibitory effect of GATA3 via repression of tissue microenvironment remodelling factors such as MMP and VEGFA [68, 79]. The functions of miR-29b relevant to phenotypic plasticity in melanoma remain comparatively very poorly-defined. In a study of an IFN-γ-STAT1-miR-29 family interaction circuit, a minority of patient-derived primary melanomas were found to have markedly elevated miR-29b and miR-29a levels relative to benign nevi and normal skin, whilst metastatic samples had marginal or no elevation in miR-29a/b abundance [80]. In a small panel of melanoma cell lines an inverse relationship between miR-29b level and melanoma cell line proliferation was observed, seemingly at odds with the higher miR-29a ~ b1 cluster abundance in some primary melanomas, and our finding that high miR-29b levels are associated with a more proliferative, epithelial-like phenotype. The confluence-based assay used to determine proliferation in the former study, together with markedly higher/combinatorial doses of miR-29 mimics/inhibitor (up to 150nM) likely contribute to these different findings.

We examined LAMC1 and PPIC in detail as potential mediators of miR-29b-3p effects across our melanoma cell models. Laminins, including LAMC1, are abundant glycoproteins within basement membranes. Consistent with our findings in melanoma, LAMC1 suppression by miR-29a/b/c has been shown to influence prostate cancer cell migration and invasion [70]. PPIC, also known as cyclophilin C, is a peptidyl-prolyl *cis*-*trans* isomerase (PPIase) which influences protein folding. PPIases are substrates for cyclosporin A and may be secreted in response to cyclosporin exposure; PPIC plays a key role in endoplasmic reticulum redox homeostasis, together with PPIB [81]. Little is known about PPIC in cancer, however PPIA and another PPIase PIN1 have been shown to interact with key growth and signalling proteins including cyclin D1, CDK10, cdc27, and PLK1, with diverse downstream effects on MAPK signalling [82–85]. Further, PIN1 participates in EMT processes within drug-resistant breast cancer [86], and drives invasiveness and tumorigenicity of A375 melanoma cells in murine models [87]. PPIC has been used as a marker of circulating tumour cells (CTCs) in epithelial ovarian cancer [88]. Breast cancer studies suggest a more complicated network of extracellular matrix-mitogenic signalling interactions involving PPIC, osteopontin, CD147 and AKT [89], warranting further exploration in the melanoma setting given prior studies demonstrating that CD147 is a clear driver of melanoma cell proliferation and metastasis in murine models [90].

The inability of LAMC1 and/or PPIC siRNA to recapitulate the effects of a miR-29b-3p mimic within the collagen spheroid assay (Fig. 5d) is likely attributable to several aspects of miR biology. Firstly, miRs have multiple potential targets but only a subset of this repertoire will be active in a given cell; for example, a miR cannot post-transcriptionally regulate a target gene that is not expressed. Secondly, the absolute abundances of miR and target gene influence the likelihood of a physical interaction, which is a pre-requisite for a regulatory interaction. Importantly, the abundances of both miR and targets may vary over time, thus creating a “moving network” of interactions. Finally, the magnitude of effect exerted by a miR on a target gene is not easily predicted and may influence the functional outcome. Taken together, these factors imply that the overall functions of a miR may be difficult to predict by perturbing any single target in isolation.

Individual miR-29b family members display variable expression patterns across tumour types and tumour stage, despite all ultimately coding for mature miRs with equivalent target repertoires [71, 72]. This apparently independent regulation of expression of miRs from distinct genomic locations suggests that coordination of miR function may be intricately linked to the expression of other genomic features, possibly linked by transcription factor binding sites, co-location, and local epigenetic chromatin modification, all of which may combine with other regional genomic effects to produce distinct biological consequences.

## Conclusions

Two key features of micro-RNA biology are their ability to target and downregulate a large number of mRNAs [27], and the ability of many micro-RNAs to target a single mRNA, resulting in a network architecture for regulatory interactions [26]. We studied pairwise associations in a large panel of melanoma cell lines informed by empirical phenotypic features. A defining feature of this analysis is the examination of mutual information together with Pearson’s correlation, allowing us to identify strong associations which may are not necessarily linear, before the principled inclusion of sequence-based miR-mRNA predicted interactions. Our approach should provide a useful framework to guide experimental work and elucidate the role of miRs in controlling cell phenotype across a number of cancers.

Our findings implicate miR-29b as a regulator of cellular phenotype in melanoma through proteins including LAMC1 and PPIC within a network of post-transcriptionally regulated genes. It is likely, however, that a complex interplay between these and other as yet undetermined miR-29b targets combine to define the overall cellular behaviours observed. Future work will focus on predicting and modelling more complex transcriptome-wide interactions. Such studies will need to address synchronous and competitive interactions, as well as sequential multi-step interactions such as those mediated by transcription factors. Experimentally validating such networks may be technically challenging, particularly under conditions simulating physiological levels of miR and mRNA transcript abundance. Nonetheless, combinatorial perturbations informed by computational modelling will enhance our understanding of summative biological effects relevant to key cell state transitions in cancer, as typified by EMP.

## Methods

### Overview

**Fig. 6:**
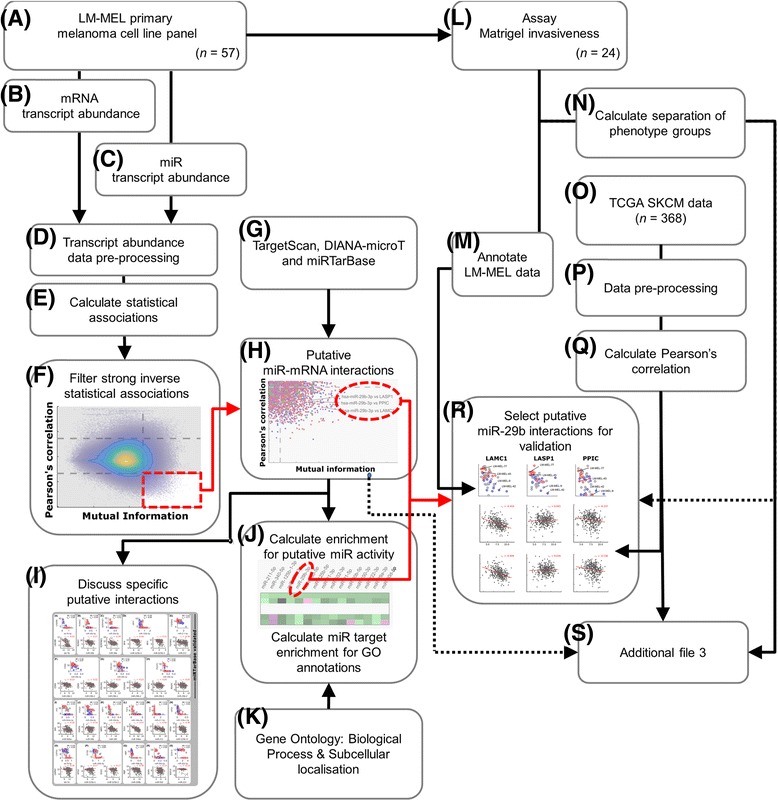
Computational workflow overview. **a** 57 melanoma cell lines derived (**b**) underwent mRNA and **c** miR transcript abundance profiling. **d** The data were processed and (**e**) measures of statistical association were calculated between miR and mRNA transcripts, across the LM-MEL panel data. **f** Strong, negative (inverse) associations (Fig. 1a) were filtered and (**g**) predicted interactions from TargetScan and/or DIANA-microT, and validated interactions from miRTarBase (**h**) were matched to provide a list of putative melanoma-relevant associations (Fig. 1b). **i** A number of interesting putative relationships were examined in further detail (Fig. 2). Enrichment of (**j**) miR-target associations within our list, and (**k**) Gene Ontology annotations associated with epithelial-mesenchymal plasticity or melanogenesis/pigmentation were calculated (Fig. 3a). **l** Independent phenotypic (invasiveness) data were used to (**m**) annotate results and (**n**) identify putative miR-mRNA relationships which separated invasiveness groups. **o** Samples from the TCGA melanoma data were (**p**) processed and matched allowing (**q**) Pearson’s correlation between miRs and mRNAs in vivo, to be calculated. **r** miR-29b-3p appeared to have a novel role regulating melanoma invasiveness, and several putative relationships were validated experimentally (Figs. 3b & 4). **s** All putative relationships are listed within Additional file 3. Please refer to the main text for details

Figure 6 provides an illustrative overview of our analytical pipeline that integrates various data sources (Additional file 1), with additional technical detail given below. For the LM-MEL melanoma cell line panel [40], published mRNA and novel miR transcript data were collected and processed (Fig. 6a-d). Statistical associations were calculated and filtered (Fig. 6e-f), before integrating the TargetScan [47–49], DIANA-microT CDS [50, 51] and miRTarBase [91] databases to identify a list of putative relationships (Fig. 6g-h). We examine and discuss putative miR-mRNA associations in Fig. 2 (Fig. 6i), then explore the relative enrichment of putative relationships for each miR, and the functional annotations of putative miR targets (Fig. 6j-k). Phenotypic invasiveness assays for 24 cell lines were used for functional annotations associated with each putative relationship (Fig. 6l-n), and TCGA melanoma samples were used to consider the potential for these associations within clinical tumour data (Fig. 6o-q).

Evidence suggested that miR-29b-3p influences melanoma invasiveness through regulating several mRNA transcripts (Fig. 6r), thus these putative relationships were experimentally investigated. All putative miR-mRNA relationships are listed in Additional file 3 (Fig. 6s).

### Computational scripts

The miR-mRNA interaction pipeline for the LM-MEL data (Fig. 6d-n, r & s) was developed using MATLAB (R2015a) and a selection of data-processing scripts were developed using R (v3.2.3; Fig. 6b, c, o & p) and python (v3.0; Fig. 6i [TCGA plots], q & r). All computational scripts developed for this project are freely available under an MIT license, from: https://github.com/uomsystemsbiology/LMMEL-miR-miner

To reproduce these results without MATLAB and extensive system configuration (e.g. installation of python packages), a Virtual Reference Environment [92] is available; however, users must accept the MATLAB Compiler Runtime Libraries License displayed during installation: https://github.com/uomsystemsbiology/LMMEL-miR-miner_reference_environment

### LM-MEL panel mRNA data

The Ludwig Melbourne melanoma (LM-MEL) panel comprised of 57 cell lines derived from mostly metastatic melanoma tumours (Fig. 6a) has previously undergone mRNA transcript abundance profiling with the Illumina HT12 beadchip microarray platform, version 4 [40]. Data were downloaded from ArrayExpress (Additional file 1) and underwent background correction, quantile normalisation and log_2_-transformation within R, retaining multiple probes as distinct observations (Fig. 6b).

### LM-MEL panel miR data

MicroRNA profiling was performed across the LM-MEL panel of cell lines by small RNA sequencing at the Australian Genome Research Facility (AGRF) on the Illumina HiSeq platform using total RNA, including small RNAs, purified from cell line pellets using the Qiagen miRNEasy isolation kit, following the manufacturer’s recommendations (Qiagen, Chadstone, Victoria, Australia). Library preparation and 5’-barcode multiplexing were performed prior to sequencing; each sample was run in 3–4 sequencing lanes as required to achieve adequate sequencing depth. Initial read quality assessment and filtering were performed by AGRF. De-multiplexed raw read data and quality scores were provided in fastq format. All reads for each sample were concatenated using the UNIX command line and collapsed to single fasta format files using the FASTX-Toolkit (v. 0.0.13) command-line tool FASTQ Collapser. Collapsed reads were processed through the miRanalyzer webserver [93] to map reads to the genome using the hg18 build of the UCSC *Homo sapiens* genome, followed by mapping of miRs to miRBase [94–99]. Raw and processed miR abundance data were deposited on the Gene Expression Omnibus (GEO; dataset GSE89438; Additional file 1). These data underwent normalisation and a log_2_-transformation was performed (Fig. 6c).

### LM-MEL phenotypic invasiveness data

Invasiveness through matrigel-coated Boyden chambers (8 μm pore size) was profiled for 24 LM-MEL cell lines (Fig. 6l); cell lines were subsequently grouped as high- or low-invasiveness (Additional file 1) and used for annotation (Fig. 6m) and further analysis (Fig. 6n).

### TCGA miR and mRNA data

Matched miR and mRNA sequencing data from TCGA skin cutaneous melanoma samples were downloaded (Additional file 1), comprised of 368 samples from 366 individual patients (Fig. 6o). R scripts were developed to use the dataset’s native directory structure for extracting and compiling normalised mature microRNA isoform read counts (including ‘star’ [*] forms), and RNAseq normalised read counts from all samples (Fig. 6p). Samples were matched across the microRNA and RNA data according to the unique TCGA patient/sample barcodes.

### LM-MEL data pre-processing

To reduce the computational burden of the statistical analysis, the LM-MEL mRNA and miR abundance data were pre-processed (Fig. 6d) to remove RNAs with a very low abundance or dynamic range, as these features can indicate a poor signal-to-noise ratio for the data. The 10^th^-percentiles of mRNA and miR abundance were identified, and any miRs or mRNAs with less than 25% of observations (i.e. across the 14/57 cell lines) above their respective thresholds were excluded. Similarly, miRs which did not have a range greater than the 90^th^ percentile of the miR abundance data and mRNA probes which did not have a range greater than 10% of the total mRNA data range, were also filtered. This pre-processing retained 198/2592 miRs (note that 916 of the miRs had no reads across any cell line) and 16482/47231 mRNA probes within the LM-MEL data.

### Statistical associations

Pearson’s correlation and mutual information were calculated between miR and mRNA abundance, across all pairwise combinations of miRs and mRNAs which passed pre-processing (Fig. 6e). The intrinsic ‘*corr*’ function within MATLAB (R2015a; Statistics and Machine Learning Toolbox) was used to calculate the Pearson’s correlation.

As noted earlier, mutual information is a measure of statistical association which is formulated such that it will tend towards zero in the case of statistical independence between the miR and mRNA abundance. Mutual information was calculated using the Java Information Dynamics Toolkit (JIDT) [43] with the ‘multivariate Kraskov 2 estimator’ (i.e. miR and mRNA transcript abundance data were treated as continuous variables and the Kraskov-Stoegbauer-Grassberger estimator was used to calculate the MI), implemented within the MATLAB Network Analysis and Inference Libraries [42]. Note that the JIDT corrects for mutual information over-estimation [46], and this subtraction can lead to negative mutual information values (Fig. 1a & b).

For the TCGA data analysed within python, correlations were calculated (Fig. 6q) using the numpy package [100] and plots (Figs. 6I & 7r) were produced using the matplotlib package [101].

Under the hypothesis that miR-mediated degradation of target mRNA transcripts would manifest as a negative association, we filtered for the top 10% of associations when ranked by mutual information (mutual information > 0.259), and the most negative 2.5% of associations when ranked by Pearson’s correlation (Pearson’s correlation < −0.330; Figs. 1a & 7f).

### Databases

Ensembl BioMart [102] was used to match identifiers between different databases as necessary. To reduce the search domain of putative interactions and filter indirect associations which may arise through the modulation of an intermediate regulatory component (e.g. a transcription factor), we filtered for predicted miR-mRNA relationships with a relatively high confidence. Specifically, we took the top 15^th^ percentile of TargetScan v7.0 (context + score < −0.286) [47–49], and/or the top 30^th^ percentile of DIANA-microT CDS (miTG-score > 0.634) [50, 51] (Fig. 6g). The miR-mRNA relationships which had been experimentally validated with ‘strong evidence’ on miRTarBase [91] (‘Luciferase reporter assay’; 'qRT-PCR' or 'Western blot') were also extracted (Fig. 6g) and used to annotate the selected statistical associations. This allowed us to identify a set of putative miR-mRNA relationships (Figs. 1b & 7 h) which may show graded levels of activity across different melanoma samples, contributing to differences in cell phenotype.

Micro-RNAs can regulate large phenotypic changes through distributed regulation of targets with a related function [27]. Annotations were downloaded from the Gene Ontology [103] database [104] (Fig. 6k) and mRNAs were matched to GO annotations using strings related to epithelial-mesenchymal plasticity (‘epith’,’mesench’) or pigmentation/melanogenesis (‘pigment’, ‘melan’; Fig. 6j, *at bottom*). GO categories were excluded if they had less than 5, or more than 500, gene members, and all categories are listed in Additional file 6.

### Invasiveness-separation metric

A metric was created to quantify the relative separation of low- and high-invasive cell line groups over the miR abundance ([miR]) versus mRNA abundance ([mRNA]) association (Fig. 6n). Calculating the centroid/geometric mean of the low- $\left({\widehat{\mu}}_{miR, low},\;{\widehat{\mu}}_{mRNA, low}\right)$ and high-invasive $\left({\widehat{\mu}}_{miR, high},\;{\widehat{\mu}}_{mRNA, high}\right)$ groups from mRNA and miR abundances, we defined:

$$ {d}_{Sep, \max }=\sqrt{{\left( \max \left(\left[ mRNA\right]\right)- \min \left(\left[ mRNA\right]\right)\right)}^2+{\left( \max \left(\left[miR\right]\right)- \min \left(\left[miR\right]\right)\right)}^2} $$

$$ {d}_{Sep, norm}=\frac{\sqrt{{\left({\widehat{\mu}}_{nRNA, high}-{\widehat{\mu}}_{mRNA, low}\right)}^2+{\left({\widehat{\mu}}_{miR, high}-{\widehat{\mu}}_{miR, low}\right)}^2}}{d_{Sep, \max }} $$

The percentile rank of d_Sep,norm_ was calculated for all miR-mRNA associations, where the miR and mRNA association passed the data pre-processing, and is listed with corresponding relationships within Additional file 3.

### Cell line culture

Melanoma cell lines were obtained from institutional stocks, as previously described [40]. LM-MEL-7, −9, −42, −45 and −77 were used for in vitro experiments; identity was confirmed by STR profiling and HLA-matching to the documented patient of origin. All cell lines were maintained in adherent culture incubated in 5% atmospheric CO_2_ at 37°C in RPMI 1640 media (Gibco®, Life Technologies™, Mulgrave VIC, Australia) supplemented with 10% fetal bovine serum (Sigma, St Louis MO, USA), 1% glutamine (glutaMAX™) and 1% penicillin/streptomycin (both from Gibco®, Life Technologies™).

### Transient transfections with miR inhibitor/mimic or siRNA

Cells were seeded into 6, 12 or 96 well plates as required for planned downstream assays. Transfections were performed when cells were approximately 70% confluent, using the Lipofectamine® RNAiMAX transfection reagent (Invitrogen™, Life Technologies™) at 0.3, 2 or 3 μL per well of 96, 12 or 6 well plates, respectively, diluted in OptiMEM® reduced serum medium (Gibco®, Life Technologies™) and mixed 1:1 with microRNA/siRNA construct (also in OptiMEM®) to a final volume producing a 1:5 final dilution into growth media. For microRNA transfections, cells were forward transfected with mirVana™ miRNA inhibitor negative control #1 or miRNA-29b-3p inhibitor, miRNA mimic negative control #1 or miRNA-29b-3p mimic (all Ambion™, Life Technologies™, Cat. Nos. 4464076, 4464084, 4464058, 4464066) at final concentrations as indicated in results. For gene target knockdown studies, cells were transfected with siRNA scrambled control or specific siRNAs targeting LAMC1, LASP1 or PPIC (all OriGene Trilencer-27 Human siRNA, Cat. Nos. SR30004, SR302649, SR302655, SR303664; Rockville, MD, USA), at doses indicated in results. For spheroid assays, cells were reverse transfected using identical reagent mixtures at the time of seeding onto agar.

### Real time-PCR

Total RNA was collected from treated/untreated cell pellets using RNeasy (standard gene qPCR only) or miRNeasy (total RNA including small RNA) kits (Qiagen, Melbourne, Victoria, Australia) and cDNA formed using the high-capacity cDNA reverse transcription kit (Applied Biosystems™, Life Technologies™, Cat. No. 4368814) or TaqMan® microRNA reverse transcription kit (Cat. No. 4366596), respectively, according to the manufacturer’s protocol. MicroRNA qPCR was performed using TaqMan® Universal PCR Master Mix, no AmpErase® UNG (Cat. No. 4324018) and TaqMan® microRNA assays (all Cat. No. 4427975) specific for hsa-miR-211 (ID 000514), hsa-miR-125b-1* (ID 002378), hsa-miR-221* (ID 002096), hsa-miR-9 (ID 000583), hsa-miR-23b (ID 000400), hsa-miR-29b (ID 000413), hsa-miR-222 (ID 002276), hsa-let-7a (ID 000377), or controls RNU44 (ID 001094) and RNU24 (ID 001001). Target gene qPCR was performed using SensiFAST™ SYBR® Lo-ROX mastermix (Bioline, Alexandria, NSW, Australia) with PCR primers designed as per Table AF5.1 in Additional file 5.

Data were collected as ∆∆CT with melt curve inspection using the ViiA™ 7 Real-Time PCR System and accompanying software (Applied Biosystems™, Life Technologies™) based on technical triplicates. Mean CT from 2 to 3 biological replicates was expressed as copies per 10,000 copies of a reference gene (β-actin, RNU24 or RNU44).

### In vitro proliferation assays

Cells were seeded at 5000 cells per well in flat-bottomed 96-well tissue culture plates prior to transfection with microRNA or siRNA agents as described above. Cellular proliferation was measured at baseline (i.e. pre-transfection) and at 24 and 72 h following transfection by incubation for 1.5 h in MTS reagent (CellTiter 96® AQueous One Solution Cell Proliferation Assay, Promega, Madison WI, USA) diluted 1 in 6 in culture media, followed by measurement of the absorbance at 490 nm and background correction using a no-cell control.

### Low-density seeding assays

Transfected cells were re-harvested and plated in 6-well tissue-culture treated plates at a density of 2000 cells per well, in 2 mL culture medium. Cells were monitored visually each day and allowed to grow for 21 days prior to fixation in 2% paraformaldehyde (Electron Microscopy Sciences, Hatfield, PA, USA) and staining with 0.01% crystal violet/10% ethanol. Plates were imaged in the 680 nm infrared channel using the Odyssey scanner (LI-COR® Biosciences, Lincoln, NE, USA) prior to subsequent quantitation of cellular outgrowth using the Colony Area plugin for ImageJ (version 1.47) [105, 106].

### Western blotting

Protein was harvested from cells 96 h following transient transfection with miR-29b agents or specific siRNAs against LAMC1, LASP1 or PPIC. Cell lysates were prepared in 100 μL of RIPA buffer (Thermo Scientific™ Pierce™, Cat. No. 89900) containing the manufacturer’s recommended concentrations of PhosSTOP™ phosphatase inhibitor and cOmplete™ ULTRA EDTA-free protease inhibitor (Roche Diagnostics GmbH, Mannheim, Germany), incubated for 30 min at 4°C prior to manual scraping of cells/lysates. Protein concentrations were estimated using the BCA method (Thermo Scientific™ Pierce™ BCATM Protein Assay Kit, Cat#23225) as per the manufacturer’s directions. Samples were immediately adjusted to uniform final concentrations by the addition of extra lysis buffer as required, prior to the addition of NuPAGE® LDS Sample Buffer (4×) and NuPAGE® sample reducing agent (10×) (both from Novex®, Life Technologies, Cat#NP008 and Cat#NP009). Samples were electrophoresed on NuPAGE® Novex® Bis-Tris 4–12% pre-cast gels prior to transfer to nitrocellulose membrane using the iBlot® Dry Blotting System (Invitrogen™, Life Technologies, Mulgrave, Victoria, Australia). Membranes were blocked in Odyssey Blocking Buffer (LI-COR® Biosciences) for 1 h at room temperature before probing for LAMC1 (apparent MW 178 kDa) using the rabbit anti-laminin gamma 1 monoclonal antibody RabMab [EPR8217] (Abcam, Cat. No. ab134059; Melbourne, Victoria, Australia) at 1:1000, for LASP1 (apparent MW 30 kDa) using the rabbit polyclonal anti-LASP1 – N-terminal antibody (Abcam, Cat. No. ab191022) at 1:1000, or for PPIC using the rabbit monoclonal anti-PPIC – C-terminal antibody RabMab [EPR15355] (Abcam, Cat. No. ab184552) at 1:10,000. Loading was controlled to β-Actin (apparent MW 45 kDa) using the mouse monoclonal anti-β-Actin (8H10D10) antibody (Cell Signaling Technology, Cat. No. 3700; Danvers, MA, USA) at 1:3000. Membranes were incubated with primary antibodies for 2 h at room temperature in a 1:1 mixture of PBS containing 0.05% tween-20 (PBST) and Odyssey Blocking Buffer. Bands were visualised by staining with IRDye® 680RD goat anti-mouse IgG and IRDye® 800CW goat anti-rabbit IgG secondaries at 1:40,000 and 1:20,000 respectively (LI-COR®, Prod. Nos. 926–68070 and 926–32211) for 1 h at room temperature. Air-dried membranes were scanned on the LI-COR® ODYSSEY® Infrared Imaging System and densitometry performed in the Image Studio™ Lite Version 5.0 software (LI-COR®). Densitometry results were normalized to β-Actin and expressed relative to the appropriate control treatment.

### Undirected radial migration assays

Transfected cells were re-harvested, counted and delivered at 40,000 cells per well of 96 well optical-bottom plates (Nunc, ThermoFisher Scientific) fitted with Oris™ Cell Migration Assay stoppers (Platypus Technologies, Madison, WI, USA). Cells were allowed to adhere overnight prior to removal of stoppers, leaving a 2 mm diameter detection zone in the centre of each well. After changing to fresh growth media, cells were allowed to migrate for 24–48 h prior to fixation in 2% paraformaldehyde and staining with 0.01% crystal violet/10% ethanol.

### Spheroid collagen invasion assays

Collagen-implanted spheroid invasion was performed based on previously-described methods [107]. Spheroids were prepared by seeding 150,000 cells in 2 mL of growth medium (with required transfection reagents) into wells of six well plates on top of pre-formed 0.8% agarose base-layers (Sigma, Cat. No. A9539) diluted from sterilised 3.2% agarose stock with growth medium. After 48 h incubation, spheroids were harvested by gentle aspiration, transferred to sterile collection tubes and allowed to settle by gravity for 30 min prior to manual aspiration of overlying media. Neutralised collagen was prepared from 5 mg/mL bovine collagen type I stock (Trevigen Cultrex®, Cat. No. 3442-050-01, Gaithersberg, MD, USA) by dilution 1:1 with 2× growth medium (2× RPMI 1640, 20% FBS) and pH adjusted by dropwise addition of sterile 1 M NaOH. Acellular collagen base layers were prepared by aliquoting 300 μL of neutralised collagen into wells of a 24 well plate and allowed to gel at room temperature for 30 min. Spheroids were then gently resuspended in 500 μL of neutralised collagen diluted 2:1 with 1× growth medium (final collagen concentration 1.67 mg/mL) and overlaid onto base layers and allowed to gel for 10 min at room temperature before returning to the incubator. Once completely gelled, collagen was overlaid with a further 500 μL of growth media. Spheroids were allowed to invade for 24 h prior to fluorescent viability staining with calcein-AM (live) and EthD-III (dead) using the Live/Dead Cell Staining Kit II (PromoKine, Cat. No. PK-CA707-30002, PromoCell GmbH, Heidelberg, Germany) before imaging.

## Additional files

Additional file 1: Data and Computational Tools (.docx) – Contains **Table AF1.1**, which lists detailed information on the data and computational tools used in this study. (DOCX 14 kb)
Additional file 2: Table (.csv) of matrigel invasiveness (classified as invasive/non-invasive) for LM-MEL cell lines which is read into the computational scripts. For details on the experimental protocol please refer to Methods/LM-MEL phenotypic invasiveness data. (CSV 273 bytes)
Additional file 3: Table (.csv) output from the computational scripts containing (1041) putative relationships with a strong negative statistical association and support from TargetScan and/or DIANA-microT, or mirTarBase (i.e. the full list of associations from Fig. 1b). mRNA Transcripts which have been annotated with a role in pigmentation or EMP via their GO category (from Fig. 1c) are labelled. mRNA transcripts which have been previously implicated in phenotypic switching in melanoma are labelled [3]. (CSV 77 kb)
Additional file 4: miRNA-mRNA Interactions of Interest (.docx) – Contains: **Table AF4.1**, which lists miRNA-mRNA interactions identified from our analysis that have been previously validated in human cellular contexts. **Table AF4.2**, which lists novel putative miRNA-mRNA interactions that involve mRNA transcripts implicated in melanoma EMP behaviours and/or invasiveness. (DOCX 86 kb)
Additional file 5: Supporting Experimental Data (.pdf) – Contains: **Table AF5.1**, which lists the PCR primers used. **Figure AF5.1** which displays baseline expression for genes of interest in a subset of LM-MEL melanoma cell lines using qPCR. **Figure AF5.2**, which displays siRNA transfection efficiency; **Figure AF5.3**, which displays additional qPCR results showing the effects of miR-29b mimic/inhibitor transfection. **Figure AF5.4 & AF5.5**, which display the effects of miR-29b mimic/inhibitor transfection on LM-MEL-77 cell proliferation and outgrowth. **Figure AF5.6**, which displays information to aid the reader’s interpretation of melanoma spheroid data. (PDF 3182 kb)
Additional file 6: Table (.csv) of Gene Ontology (GO) database terms used for the enrichment analysis of ‘epithelial-mesenchymal plasticity’ (EMP) and pigmentation processes in Fig. 3a, together with the number of genes within each category. As detailed in Methods/Databases, terms were identified by string matching and GO terms were excluded if they had less than five or more than 500 member genes. (CSV 5 kb)

## Abbreviations

CTCs: Circulating tumour cells
EMP: Epithelial-mesenchymal plasticity
EMT: Epithelial-to-mesenchymal (like) transition
LAMC1: Laminin, γ1 (Entrez Gene ID 3915)
LASP1: LIM and SH3 Protein 1 (Entrez Gene ID 3927)
LM-MEL: Ludwig Melbourne melanoma cell line
miR: microRNA
miR-29b: hsa-miR-29b-3p (miRBase Accession: MIMAT0000100)
PPIC: Peptidylprolyl Isomerase C (Cyclophilin C; Entrez Gene ID 5480)
TCGA: The Cancer Genome Atlas
