# Supplementary material for: Systems analysis identifies miR-29b regulation of invasiveness in melanoma
Source: Mol Cancer. 2016 Nov 16;15:72. doi: 10.1186/s12943-016-0554-y (PMC5112703; doi:10.1186/s12943-016-0554-y)
Supplement: Additional file 1: — Data and Computational Tools (.docx) – Contains Table AF1.1, which lists detailed information on the data and computational tools used in this study. (DOCX 14 kb) [file 12943_2016_554_MOESM1_ESM.docx]

***Additional file 1 – Data and Computational Tools***

**Table AF1.1: Data and Tools used in this study.**

| **mRNA and miR Transcript Abundance Data** |
| --- |
| (**A**) LM-MEL mRNA data  ArrayExpress accession: E-MTAB-1496 |
| (**B**) LM-MEL miR data  Gene Expression Omnibus data series GSE89438  Available from: <https://www.ncbi.nlm.nih.gov/geo/> |
| (**C**) The Cancer Genome Atlas (TCGA) skin cutaneous melanoma (SKCM) data  Available from: <https://tcga-data.nci.nih.gov/tcga/>  Accessed: 28/08/2014 |
| **Databases** |
| (**D**) TargetScan (v7.0) {Friedman, 2009 #46;Grimson, 2007 #47;Lewis, 2005 #48}  Available from: <http://targetscan.org/>  Accessed: 19/08/2015 |
| (**E**) DIANA-microT CDS {Paraskevopoulou, 2013 #49;Reczko, 2012 #50}  Available from: [http://www.microrna.gr/microT-CDS](http://diana.imis.athena-innovation.gr/DianaTools/index.php?r=microT_CDS/index)  **NB**: requires account creation with DIANA Tools  Accessed: 23/01/2016 |
| (**F**) miRTarBase (v6.1) {Chou, 2016 #124}  Available from: <http://mirtarbase.mbc.nctu.edu.tw/>  Accessed: 11/01/2016 |
| (**G**) Gene Ontology {Ashburner, 2000 #136;Gene Ontology, 2015 #137}  Available from: <http://geneontology.org/>  Accessed: 28/10/2015 |
| (**H**) miRBase (v. 18) {Griffiths-Jones, 2010 #127;Griffiths-Jones, 2006 #128;Griffiths-Jones, 2006 #129;Griffiths-Jones, 2008 #130;Kozomara, 2014 #131;Kozomara, 2011 #132}  Available from: <http://www.mirbase.org/>  Accessed: |
| (**I**) Ensembl BiomaRt {Yates, 2016 #135}  Available from: <http://ensembl.org/biomart/martview>  Accessed: 13/05/2015 |
| **Tools** |
| (**I**) FASTX-Toolkit (v. 0.0.13)  Available from: <http://hannonlab.cshl.edu/fastx_toolkit/download.html>  Accessed: 11/01/2014 |
| (**J**) miRanalyzer (web-server) {Hackenberg, 2011 #126}  Available from: <http://bioinfo2.ugr.es/miRanalyzer/miRanalyzer.php>  Accessed: 7/01/2014 - 16/01/2014 |
