# Supplementary material for: Systems analysis identifies miR-29b regulation of invasiveness in melanoma
Source: Mol Cancer. 2016 Nov 16;15:72. doi: 10.1186/s12943-016-0554-y (PMC5112703; doi:10.1186/s12943-016-0554-y)
Supplement: Additional file 4: — miRNA-mRNA Interactions of Interest (.docx) – Contains: Table AF4.1, which lists miRNA-mRNA interactions identified from our analysis that have been previously validated in human cellular contexts. Table AF4.2, which lists novel putative miRNA-mRNA interactions that involve mRNA transcripts implicated in melanoma EMP behaviours and/or invasiveness. (DOCX 86 kb) [file 12943_2016_554_MOESM4_ESM.docx]

***Additional file 4: miRNA-mRNA Interactions of Interest***

**Table AF4.1: miRNA-mRNA interactions validated in human cellular contexts**

| **MicroRNA** | **Gene** | **Figure 2 panel** | **Comments** | **Reference(s)** |
| --- | --- | --- | --- | --- |
| let-7b-5p | LIN28B | A | **interaction**: mutual repression important for melanoma phenotype switching | (Zhang *et al* 2015b) |
| miR-30b-5p | RUNX2 | B | **gene**: implicated in melanoma invasiveness  **interaction**: shown in smooth muscle differentiation | (Boregowda *et al* 2014)  (Balderman *et al* 2012) |
| miR-30b-5p | SERPINE1 (PAI1) | C | **gene**: implicated in melanoma invasiveness  **interaction**: shown in gastric cancer | (Hoek *et al* 2008; Klein *et al* 2012)  (Zhu *et al* 2014) |
| miR-125b-5p | IRF4 (MUM1) | D | **gene**: target/co-factor for MITF, polymorphisms associated with pigmentation changes  **interaction**: influences myeloma cellular differentiation and survival | (Duffy *et al* 2010; Praetorius *et al* 2013)  (Gururajan *et al* 2010) |
| miR-211-5p | TGFBR2 | E | **interaction**: modulates TGF-β driven EMP | (Levy *et al* 2010)  (Moustakas and Heldin 2014) |
| miR-29b | CDK6 | F | **interaction**: downstream effector of IFNγ-induced STAT1 signalling; a subset of melanomas have higher miR-29a/b levels | (Schmitt *et al* 2012) |
| miR-29b | COL4A1 | G | **interaction**: not shown in melanoma | (Plaisier *et al* 2012; Steele *et al* 2010) |
| miR-29b | PDGFC | H | **interaction**: not shown in melanoma | (Chou *et al* 2013) |

**Table AF4.2: Novel putative miRNA-mRNA interactions of relevance to melanoma EMP behaviours and/or invasiveness.**

| **MicroRNA** | **Gene Symbol** | **Fig. 2 panel** | **Gene functions** | **Reference(s)** |
| --- | --- | --- | --- | --- |
| miR-500a-3p | AXL | I | breast cancer EMP  high expression in melanoma associated with:  - drug resistance;  - EMP and resistance to checkpoint inhibitor immunotherapy;  - high motility and invasive potential and lower MITF expression | (Asiedu *et al* 2014; Gjerdrum *et al* 2010)      (Muller *et al* 2014)    (Hugo *et al* 2016)  (Sensi *et al* 2011) |
| miR-30b-5p | FOXD1 | J | Forkhead box transcription factors implicated in EMP;  FOXD1 primarily studied in kidney development and healing | (Chaffer *et al* 2007)  (Gomez and Duffield 2014) |
| miR-185-5p | NRP1 | K | implicated in EMP for numerous cancers  neural crest delamination and migration processes that are recapitulated by invasive melanoma | (Adham *et al* 2014; Chu *et al* 2014; Glinka *et al* 2012)  (Theveneau and Mayor 2012)  (Bailey *et al* 2012; Ruffini *et al* 2013). |
| miR-146a-5p  miR-211-5p | TCF4 | L  M | central to EMP, complexes with β-catenin  implicated in melanoma phenotype switching | (Hugo *et al* 2007)  (Eichhoff *et al* 2011) |
| miR-125b-1-3p | HPS4 | N | melanosome formation;  suppression observed in WNT5A/PKC-mediated EMT in melanoma | (Suzuki *et al* 2002)  (Dissanayake *et al* 2007) |
| let-7e-5p | CAPN3 | O | pro-apoptotic in melanoma  downregulated during HIF1α-dependent phenotype switching in hypoxia (partly mediated via HIF1α targeting of MITF) | (Moretti *et al* 2009)  (Widmer *et al* 2013) |
| miR-125b-5p | GYG2 | P | upregulated in metastases and some primary tumours of colorectal and gastric cancers | (Kleivi *et al* 2007)  (Zhang *et al* 2015a) |
| miR-199b-5p | MBP | Q | substrate of PAK1 acting downstream of CDC42 and RAC; induction associated with trans-differentiation of melanoma to a glial cell phenotype | (Slutsky *et al* 2003) |

**References**

Adham SA, Al Harrasi I, Al Haddabi I, Al Rashdi A, Al Sinawi S, Al Maniri A, Ba-Omar T, Coomber BL. 2014. Immunohistological insight into the correlation between neuropilin-1 and epithelial-mesenchymal transition markers in epithelial ovarian cancer. J Histochem Cytochem 62(9):619-31.

Asiedu MK, Beauchamp-Perez FD, Ingle JN, Behrens MD, Radisky DC, Knutson KL. 2014. AXL induces epithelial-to-mesenchymal transition and regulates the function of breast cancer stem cells. Oncogene 33(10):1316-24.

Bailey CM, Morrison JA, Kulesa PM. 2012. Melanoma revives an embryonic migration program to promote plasticity and invasion. Pigment Cell Melanoma Res 25(5):573-83.

Balderman JA, Lee HY, Mahoney CE, Handy DE, White K, Annis S, Lebeche D, Hajjar RJ, Loscalzo J, Leopold JA. 2012. Bone morphogenetic protein-2 decreases microRNA-30b and microRNA-30c to promote vascular smooth muscle cell calcification. J Am Heart Assoc 1(6):e003905.

Boregowda RK, Olabisi OO, Abushahba W, Jeong BS, Haenssen KK, Chen W, Chekmareva M, Lasfar A, Foran DJ, Goydos JS et al. . 2014. RUNX2 is overexpressed in melanoma cells and mediates their migration and invasion. Cancer Lett 348(1-2):61-70.

Chaffer CL, Thompson EW, Williams ED. 2007. Mesenchymal to epithelial transition in development and disease. Cells Tissues Organs 185(1-3):7-19.

Chou J, Lin JH, Brenot A, Kim JW, Provot S, Werb Z. 2013. GATA3 suppresses metastasis and modulates the tumour microenvironment by regulating microRNA-29b expression. Nat Cell Biol 15(2):201-13.

Chu W, Song X, Yang X, Ma L, Zhu J, He M, Wang Z, Wu Y. 2014. Neuropilin-1 promotes epithelial-to-mesenchymal transition by stimulating nuclear factor-kappa B and is associated with poor prognosis in human oral squamous cell carcinoma. PLoS One 9(7):e101931.

Dissanayake SK, Wade M, Johnson CE, O'Connell MP, Leotlela PD, French AD, Shah KV, Hewitt KJ, Rosenthal DT, Indig FE et al. . 2007. The Wnt5A/protein kinase C pathway mediates motility in melanoma cells via the inhibition of metastasis suppressors and initiation of an epithelial to mesenchymal transition. J Biol Chem 282(23):17259-71.

Duffy DL, Iles MM, Glass D, Zhu G, Barrett JH, Hoiom V, Zhao ZZ, Sturm RA, Soranzo N, Hammond C et al. . 2010. IRF4 variants have age-specific effects on nevus count and predispose to melanoma. Am J Hum Genet 87(1):6-16.

Eichhoff OM, Weeraratna A, Zipser MC, Denat L, Widmer DS, Xu M, Kriegl L, Kirchner T, Larue L, Dummer R et al. . 2011. Differential LEF1 and TCF4 expression is involved in melanoma cell phenotype switching. Pigment Cell Melanoma Res 24(4):631-42.

Gjerdrum C, Tiron C, Hoiby T, Stefansson I, Haugen H, Sandal T, Collett K, Li S, McCormack E, Gjertsen BT et al. . 2010. Axl is an essential epithelial-to-mesenchymal transition-induced regulator of breast cancer metastasis and patient survival. Proc Natl Acad Sci U S A 107(3):1124-9.

Glinka Y, Mohammed N, Subramaniam V, Jothy S, Prud'homme GJ. 2012. Neuropilin-1 is expressed by breast cancer stem-like cells and is linked to NF-kappaB activation and tumor sphere formation. Biochem Biophys Res Commun 425(4):775-80.

Gomez IG, Duffield JS. 2014. The FOXD1 lineage of kidney perivascular cells and myofibroblasts: functions and responses to injury. Kidney Int Suppl (2011) 4(1):26-33.

Gururajan M, Haga CL, Das S, Leu CM, Hodson D, Josson S, Turner M, Cooper MD. 2010. MicroRNA 125b inhibition of B cell differentiation in germinal centers. Int Immunol 22(7):583-92.

Hoek KS, Eichhoff OM, Schlegel NC, Dobbeling U, Kobert N, Schaerer L, Hemmi S, Dummer R. 2008. In vivo switching of human melanoma cells between proliferative and invasive states. Cancer Res 68(3):650-6.

Hugo H, Ackland ML, Blick T, Lawrence MG, Clements JA, Williams ED, Thompson EW. 2007. Epithelial--mesenchymal and mesenchymal--epithelial transitions in carcinoma progression. J Cell Physiol 213(2):374-83.

Hugo W, Zaretsky JM, Sun L, Song C, Moreno BH, Hu-Lieskovan S, Berent-Maoz B, Pang J, Chmielowski B, Cherry G et al. . 2016. Genomic and Transcriptomic Features of Response to Anti-PD-1 Therapy in Metastatic Melanoma. Cell 165(1):35-44.

Klein RM, Bernstein D, Higgins SP, Higgins CE, Higgins PJ. 2012. SERPINE1 expression discriminates site-specific metastasis in human melanoma. Exp Dermatol 21(7):551-4.

Kleivi K, Lind GE, Diep CB, Meling GI, Brandal LT, Nesland JM, Myklebost O, Rognum TO, Giercksky KE, Skotheim RI et al. . 2007. Gene expression profiles of primary colorectal carcinomas, liver metastases, and carcinomatoses. Mol Cancer 6:2.

Levy C, Khaled M, Iliopoulos D, Janas MM, Schubert S, Pinner S, Chen PH, Li S, Fletcher AL, Yokoyama S et al. . 2010. Intronic miR-211 assumes the tumor suppressive function of its host gene in melanoma. Mol Cell 40(5):841-9.

Moretti D, Del Bello B, Cosci E, Biagioli M, Miracco C, Maellaro E. 2009. Novel variants of muscle calpain 3 identified in human melanoma cells: cisplatin-induced changes in vitro and differential expression in melanocytic lesions. Carcinogenesis 30(6):960-7.

Moustakas A, Heldin P. 2014. TGFbeta and matrix-regulated epithelial to mesenchymal transition. Biochim Biophys Acta 1840(8):2621-34.

Muller J, Krijgsman O, Tsoi J, Robert L, Hugo W, Song C, Kong X, Possik PA, Cornelissen-Steijger PD, Foppen MH et al. . 2014. Low MITF/AXL ratio predicts early resistance to multiple targeted drugs in melanoma. Nat Commun 5:5712.

Plaisier CL, Pan M, Baliga NS. 2012. A miRNA-regulatory network explains how dysregulated miRNAs perturb oncogenic processes across diverse cancers. Genome Res 22(11):2302-14.

Praetorius C, Grill C, Stacey SN, Metcalf AM, Gorkin DU, Robinson KC, Van Otterloo E, Kim RS, Bergsteinsdottir K, Ogmundsdottir MH et al. . 2013. A polymorphism in IRF4 affects human pigmentation through a tyrosinase-dependent MITF/TFAP2A pathway. Cell 155(5):1022-33.

Ruffini F, D'Atri S, Lacal PM. 2013. Neuropilin-1 expression promotes invasiveness of melanoma cells through vascular endothelial growth factor receptor-2-dependent and -independent mechanisms. Int J Oncol 43(1):297-306.

Schmitt MJ, Philippidou D, Reinsbach SE, Margue C, Wienecke-Baldacchino A, Nashan D, Behrmann I, Kreis S. 2012. Interferon-gamma-induced activation of Signal Transducer and Activator of Transcription 1 (STAT1) up-regulates the tumor suppressing microRNA-29 family in melanoma cells. Cell Commun Signal 10(1):41.

Sensi M, Catani M, Castellano G, Nicolini G, Alciato F, Tragni G, De Santis G, Bersani I, Avanzi G, Tomassetti A et al. . 2011. Human cutaneous melanomas lacking MITF and melanocyte differentiation antigens express a functional Axl receptor kinase. J Invest Dermatol 131(12):2448-57.

Slutsky SG, Kamaraju AK, Levy AM, Chebath J, Revel M. 2003. Activation of myelin genes during transdifferentiation from melanoma to glial cell phenotype. J Biol Chem 278(11):8960-8.

Steele R, Mott JL, Ray RB. 2010. MBP-1 upregulates miR-29b that represses Mcl-1, collagens, and matrix-metalloproteinase-2 in prostate cancer cells. Genes Cancer 1(4):381-387.

Suzuki T, Li W, Zhang Q, Karim A, Novak EK, Sviderskaya EV, Hill SP, Bennett DC, Levin AV, Nieuwenhuis HK et al. . 2002. Hermansky-Pudlak syndrome is caused by mutations in HPS4, the human homolog of the mouse light-ear gene. Nat Genet 30(3):321-4.

Theveneau E, Mayor R. 2012. Neural crest delamination and migration: from epithelium-to-mesenchyme transition to collective cell migration. Dev Biol 366(1):34-54.

Widmer DS, Hoek KS, Cheng PF, Eichhoff OM, Biedermann T, Raaijmakers MI, Hemmi S, Dummer R, Levesque MP. 2013. Hypoxia contributes to melanoma heterogeneity by triggering HIF1alpha-dependent phenotype switching. J Invest Dermatol 133(10):2436-43.

Zhang J, Huang JY, Chen YN, Yuan F, Zhang H, Yan FH, Wang MJ, Wang G, Su M, Lu G et al. . 2015a. Whole genome and transcriptome sequencing of matched primary and peritoneal metastatic gastric carcinoma. Sci Rep 5:13750.

Zhang Z, Zhang S, Ma P, Jing Y, Peng H, Gao WQ, Zhuang G. 2015b. Lin28B promotes melanoma growth by mediating a microRNA regulatory circuit. Carcinogenesis 36(9):937-45.

Zhu ED, Li N, Li BS, Li W, Zhang WJ, Mao XH, Guo G, Zou QM, Xiao B. 2014. miR-30b, down-regulated in gastric cancer, promotes apoptosis and suppresses tumor growth by targeting plasminogen activator inhibitor-1. PLoS One 9(8):e106049.
