# Supplementary material for: Systems analysis identifies miR-29b regulation of invasiveness in melanoma
Source: Mol Cancer. 2016 Nov 16;15:72. doi: 10.1186/s12943-016-0554-y (PMC5112703; doi:10.1186/s12943-016-0554-y)
Supplement: Additional file 5: — Supporting Experimental Data (.pdf) – Contains: Table AF5.1, which lists the PCR primers used. Figure AF5.1 which displays baseline expression for genes of interest in a subset of LM-MEL melanoma cell lines using qPCR. Figure AF5.2, which displays siRNA transfection efficiency; Figure AF5.3, which displays additional qPCR results showing the effects of miR-29b mimic/inhibitor transfection. Figure AF5.4 & AF5.5, which display the effects of miR-29b mimic/inhibitor transfection on LM-MEL-77 cell proliferation and outgrowth. Figure AF5.6, which displays information to aid the reader’s interpretation of melanoma spheroid data. (PDF 3182 kb) [file 12943_2016_554_MOESM5_ESM.pdf]

Additional File 5 – Supporting Experimental Data

Table AF5.1: PCR primers.

Target	Forward Primer (5’ to 3’)	Reverse Primer (5’ to 3’)
NRP1	GCCACAGTGGAACAGGTGAT	CTATGACCGTGGGCTTTTCT
TCF4	TGAGAACCTGCAAGACACGA	GGTGTCAGGTCCTCATCGTC
CPEB1	CAAGATGTCCAGCCGAAGGA	TGGGCTCCGGACAAAGTTAC
ATP2A2	TCAAGCACACTGATCCCGTC	GCTACCACCACTCCCATAGC
ADAM19	TGATCCTGGACCTGGAGAAG	TGATCCTCCAATTTCCGTGT
CAV2	CCCTCAGCTGTCTGCACAT	TCACACTCTTCCATATTGTCTGC
LAMC1	CCAACTGTCCTACTGGCACC	CTCACAGGGCCGTTTCTACC
LASP1	AAGAAGCCCTACTGCAACGC	TGCCACTACGCTGAAACCTT
PPIC	GTTGGACGGCAAACATGTGG	ATCGAGCAGTTGGTGAGTGG
β-Actin	CTGGAACGGTGAAGGTGACA	CGGCCACATTGTGAACTTTG

Quantitative PCR Validation of mRNA transcript abundances

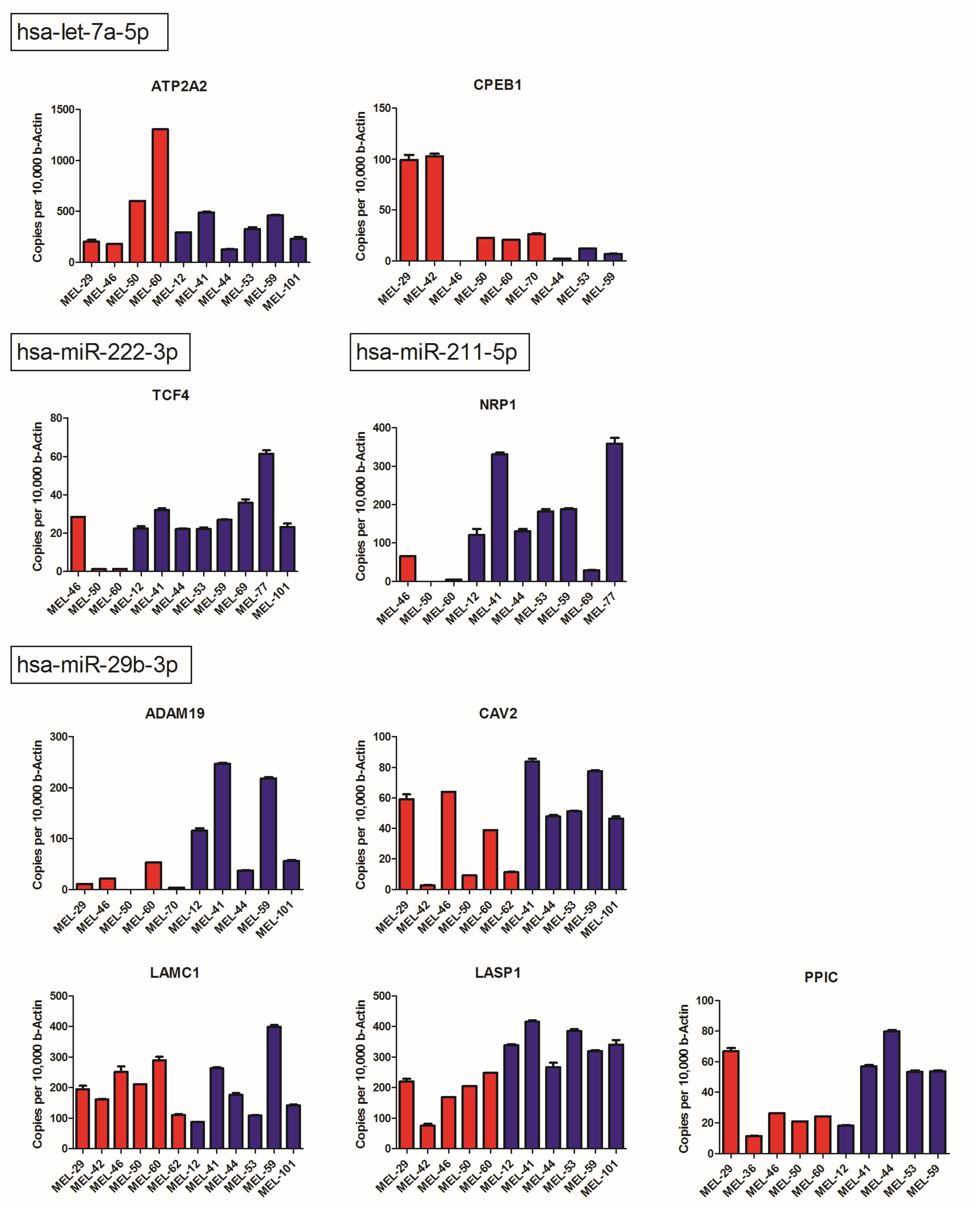

Figure AF5.1. Baseline expression of genes of interest measured by qPCR in a subset of the LM-MEL melanoma cell line panel showing both epithelial-like (red) and mesenchymal-like (blue) cell lines. Data represent mean + SEM of independent triplicates, relative to β-Actin. Genes are grouped under the micro-RNAs that were identified as putative regulators.

Testing miR transfection efficiency

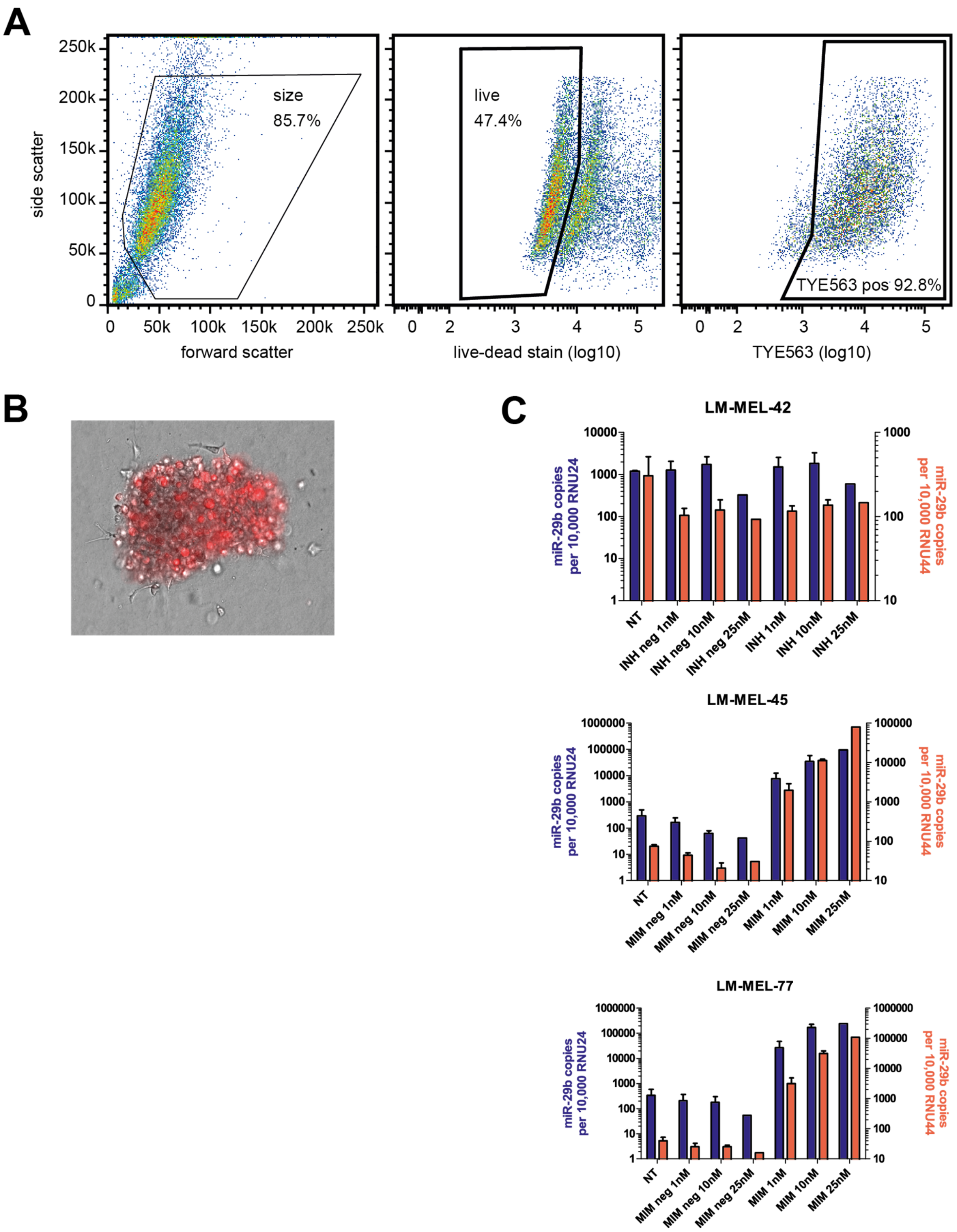

Figure AF5.2. Flow-cytometric analysis (A), and false-colour fluorescence microscopy image (B) of LM-MEL-45 cells transfected with a TYE563 fluorescently-labelled control siRNA confirmed high transfection efficiency under the conditions used in all mirVana and siRNA-transfection experiments. (C) Transfection of LM-MEL-42 with mirVana miR-29b inhibitor (INH) negative control (neg) or active agents at the doses indicated had no significant effect on PCR-amplifiable miR-29b abundance whilst equivalent miR-29b mimic (MIM) agents markedly increased detectable miR-29b levels in LM-MEL-45 and -77.

Further testing of miR mimic/inhibitor transfection effects on mRNA transcript abundance

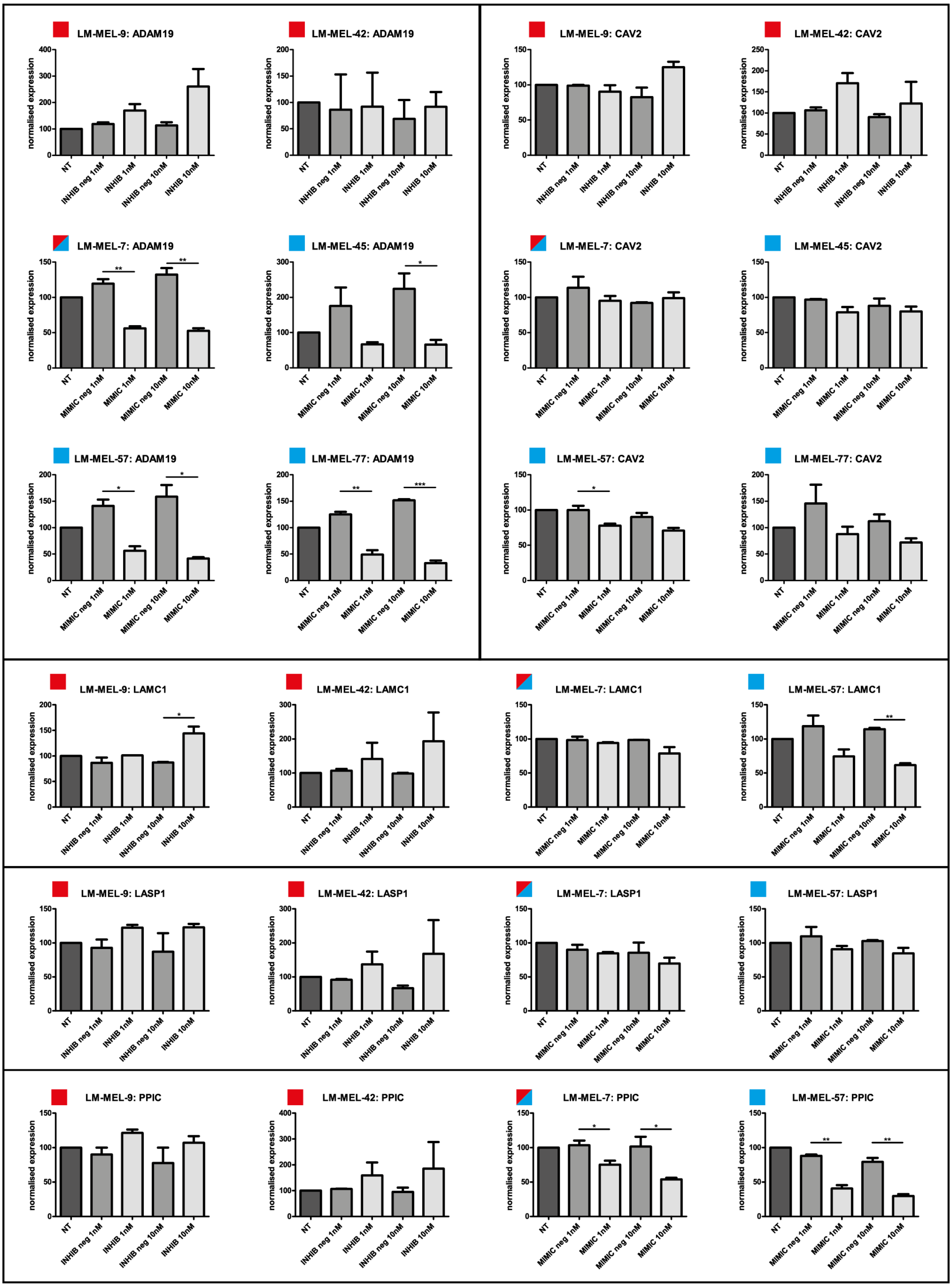

Figure AF5.3. Additional quantitative PCR results showing the effect of miR-29b mimic or inhibitor transfection, at the doses indicated, on transcript abundance of putative gene targets ADAM19, CAV2, LAMC1, LASP1 and PPIC, in additional LM-MEL cell lines. Baseline miR-29b-3p abundance is indicated by coloured boxes as follows: high (red), intermediate (red/blue), low (blue). Pairwise one-tailed t-test statistics are indicated by * $p<0.05$, ** $p<0.01$, *** $p<0.001$.

miR-29b, LAMC1, and PPIC effects on melanoma cell outgrowth and proliferation

The functional effects of miR-29b were assessed further in several in vitro assays. Perturbation of miR-29b function was achieved using a miR-29b inhibitor in LM-MEL-9 and LM-MEL-42 (E-like, high baseline levels of miR-29b), a miR-29b mimic in LM-MEL-45, -57 and -77 (M-like, low-baseline miR-29b levels) and both in LM-MEL-7 (E-like, intermediate baseline miR-29b levels). No significant differences in proliferation were seen in any cell line attributable to miR-29b treatment (Figure AF8.5.4A & Figure AF5.5), however, LM-MEL-77 was evidently sensitive to the effects of the transfection agent itself.

Next, we assessed the effect of altering putative miR-29b target genes, LAMC1 and PPIC, on proliferation of LM-MEL-45. Similar to the effects of perturbing miR-29b itself, transfection of a specific siRNA for LAMC1 or PPIC had no significant effects on the proliferation of LM-MEL-45 cells relative to controls (Fig. AF5.4A).

As a surrogate measure of cellular survival in isolated colonies, the outgrowth of LM-MEL-45 following seeding at low density was determined by densitometric analysis of stained cells/colonies after growth in 6 well tissue culture plates. Transfection of cells with a miR-29b mimic immediately prior to seeding caused a dose-dependent reduction in terminal outgrowth at 21 days relative to inactive controls (Fig. AF5.4B & C). Since the miR-29b transfection was only transient in this assay, it is tempting to speculate that this effect suggests that miR-29b can regulate molecular pathways that determine long-term cellular outgrowth.

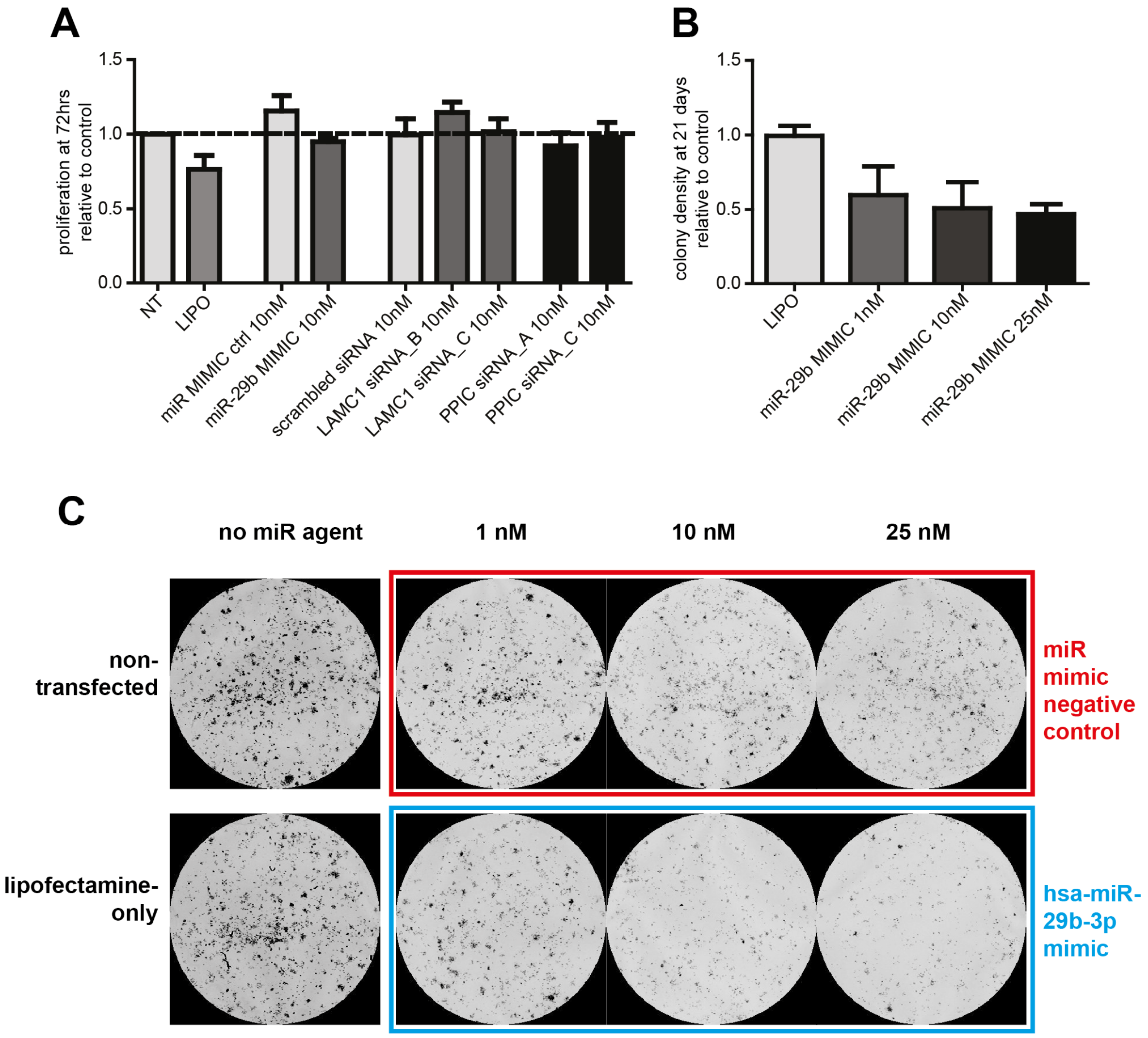

Figure AF5.4. Effect of miR-29b and specific siRNA on proliferative potential of LM-MEL-45. (**A**) Neither miR-29b-3p mimic nor siRNAs specific for LAMC1 or PPIC demonstrated any significant effect on proliferation of LM-MEL-45 cells as measured by MTS assay after 72 hours following transfection with the agents indicated. Data represent mean + SEM of 3-4 biological replicates. (**B**) Following low-density seeding at 2000 cells per well in 6-well plates, a dose-dependent trend for reduction in cellular outgrowth was observed over 21 days in LM-MEL-45 cells initially transfected with the indicated agent as measured by densitometry. (**C**) Representative infrared fluorescence images of stained culture plates showing a dose-dependent reduction in cellular outgrowth following miR-29b-3p mimic transfection. All concentrations represent final concentrations in transfection media. Data represent mean + range of cell densitometry normalised to respective controls (lipofectamine vs non-transfected, miR-29b-3p mimic vs miR-mimic negative control) from biological duplicates. NT = non-transfected, LIPO = lipofectamine (RNAiMax), ctrl = control agent.

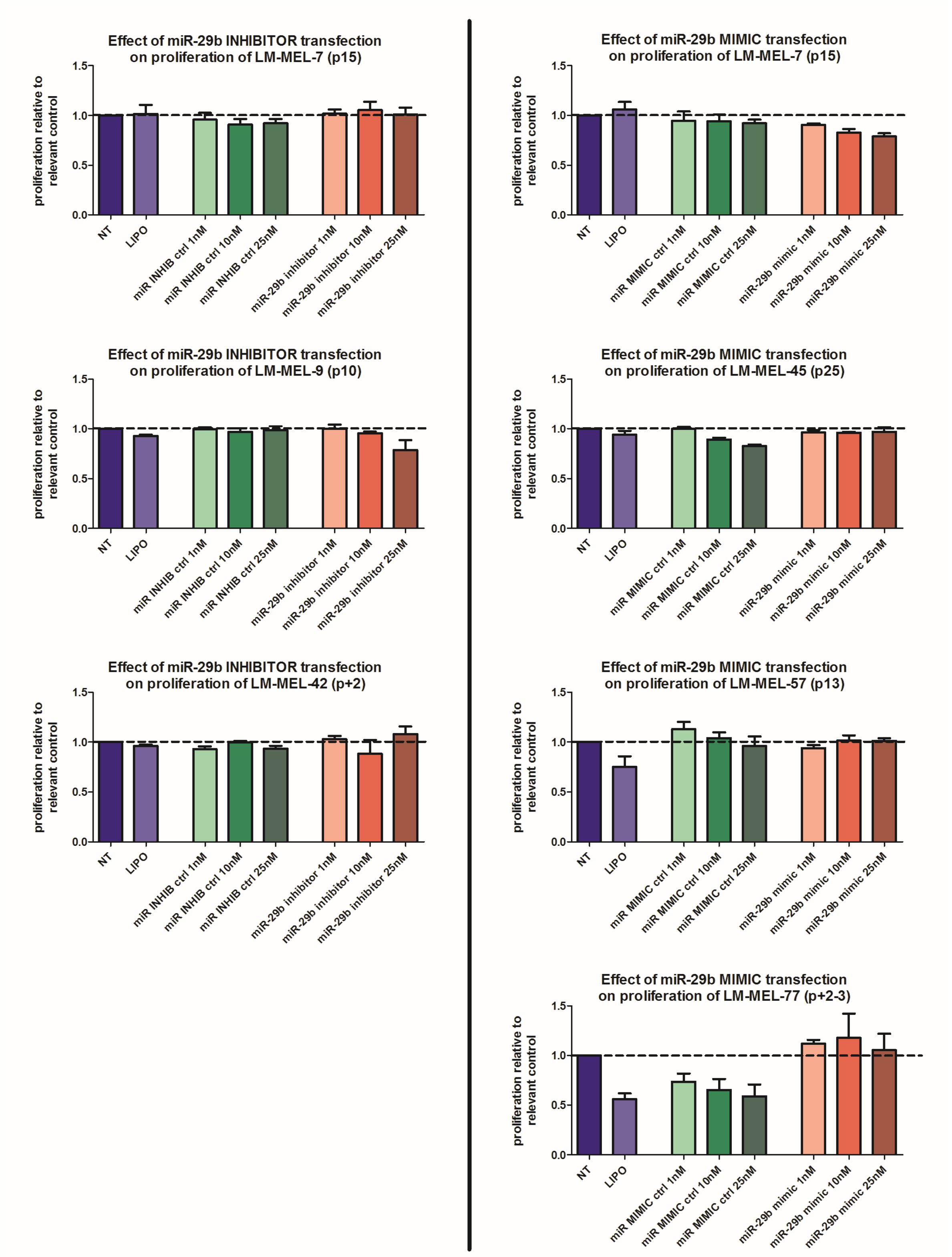

Figure AF5.5. Effect of miR-29b perturbation on proliferation of melanoma cell lines, measured by MTS assay at 72 hours following transfection with the indicated agents. Data are normalised to the respective controls (lipofectamine vs non-transfected, control agents vs lipofectamine, active agents vs control agents, dose-matched as appropriate) to indicate the specific effect of each agent. Data represent the mean + SEM of 3-4 biological replicates. NT = non-transfected, LIPO = lipofectamine-only, INHIB = inhibitor, ctrl = control agent.

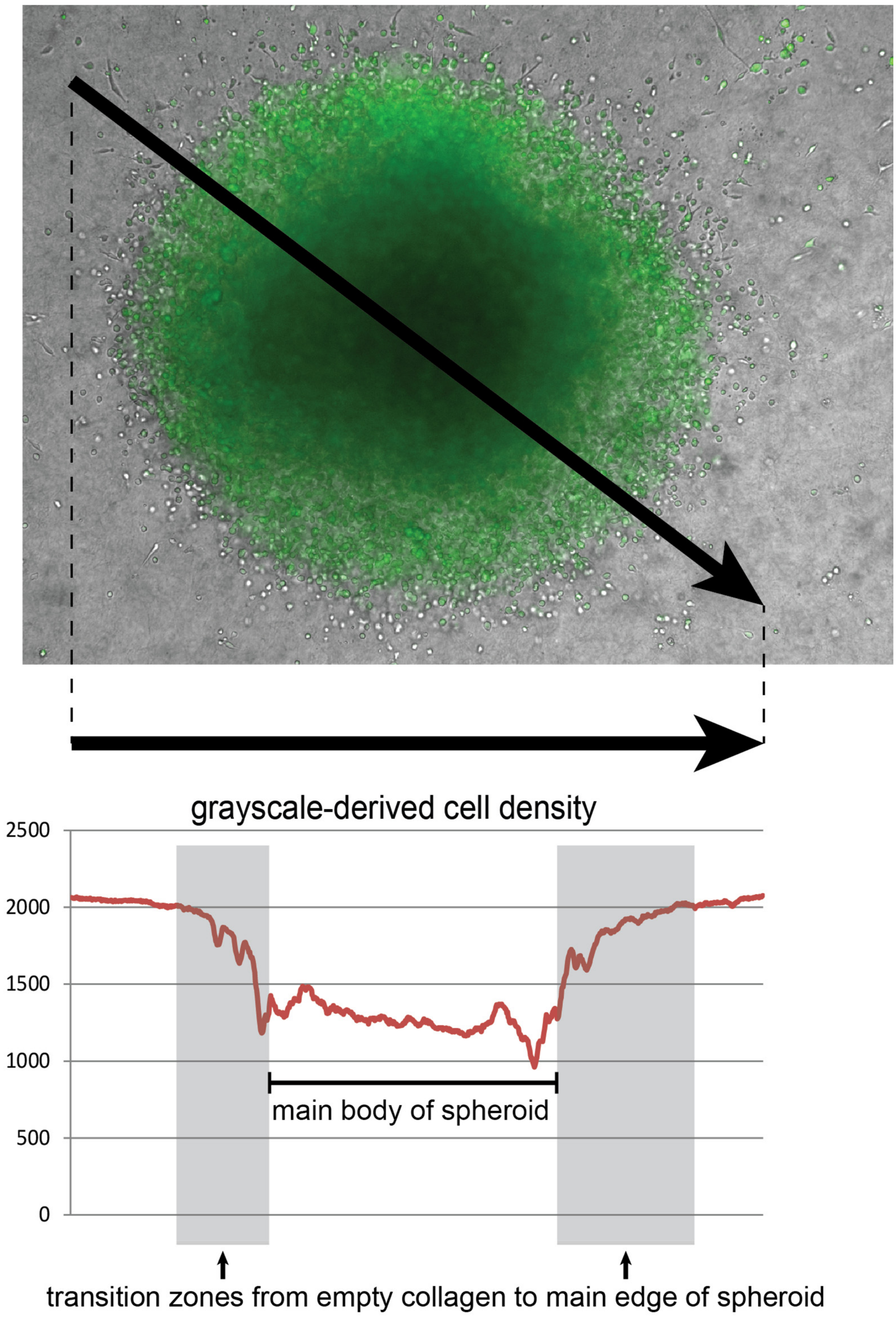

Figure AF5.6. Assessment of cross-sectional spheroid cell density. Using grey-scaled dark-background fluorescence images of viable-cell stained spheroids (here false-coloured in green), signal intensity was measured across a transverse diameter extending into surrounding acellular collagen matrix (large black arrow). The 'abruptness' of the transition from empty collagen to dense body of spheroid (transition zones, lower panel) provides a measure of invasiveness. Within the dense spheroid core, lower apparent cell densities (relative to the edges) may be observed due to incomplete penetrance of fluorescence signal.
